# Programmed Minichromosome Elimination as a Mechanism for Somatic Genome Reduction in *Tetrahymena thermophila*

**DOI:** 10.1371/journal.pgen.1006403

**Published:** 2016-11-02

**Authors:** Chih-Yi Gabriela Lin, I-Ting Lin, Meng-Chao Yao

**Affiliations:** 1 Institute of Molecular Biology, Academia Sinica, Taipei, Taiwan; 2 Genome and Systems Biology Degree Program, National Taiwan University, Taipei, Taiwan; Princeton University, UNITED STATES

## Abstract

The maintenance of chromosome integrity is crucial for genetic stability. However, programmed chromosome fragmentations are known to occur in many organisms, and in the ciliate *Tetrahymena* the five germline chromosomes are fragmented into hundreds of minichromosomes during somatic nuclear differentiation. Here, we showed that there are different fates of these minichromosomes after chromosome breakage. Among the 326 somatic minichromosomes identified using genomic data, 50 are selectively eliminated from the mature somatic genome. Interestingly, many and probably most of these minichromosomes are eliminated during the growth period between 6 and 20 doublings right after conjugation. Genes with potential conjugation-specific functions are found in these minichromosomes. This study revealed a new mode of programmed DNA elimination in ciliates similar to those observed in parasitic nematodes, which could play a role in developmental gene regulation.

## Author Summary

Chromatin diminution was first discovered in the parasite *Parascaris univalens* by T. Boveri more than one century ago. This process eliminates a large proportion of the genome accompanying germline-soma differentiation during development. Ciliates also carry out DNA elimination that have been studied in depth. During *Tetrahymena* somatic nuclear development, thousands of internal sequences are eliminated, and the 5 chromosomes are fragmented into hundreds of minichromosomes. Previous studies suggested that 1/3 of genome was deleted by the elimination of internal sequences. Here, we showed that the loss of minichromosomes also contributes to programmed DNA elimination in this ciliate. We found that these minichromosomes are eliminated during growth soon after conjugation but before sexual maturation. They are able to increase their copies by endoduplication in late conjugation stages and express genes they contain, but are later lost at similar time periods in the vegetative stage, possibly involving replication/segregation failure and/or active degradation. The elimination of minichromosomes in *Tetrahymena* follows different mechanisms to reach a similar consequence as in nematodes for somatic nuclear differentiation.

## Introduction

Eukaryotic chromosomes are the structural bases of inheritance, and contain key components such as centromeres and telomeres to maintain stability and facilitate transmission. Loss of chromosome integrity causes severe problems including tumorigenesis [1–3]. In order to maintain chromosome stability, cells initiate several responses to DNA damages, such as cell cycle arrest, apoptosis, and DNA repair [4, 5]. Double-stranded DNA breakage (DSB) is a major form of DNA damage, which is often repaired by homologous recombination, nonhomologous end-joining or end-healing (*de novo* telomere addition). End-healing is mutagenic, but interestingly, organisms have evolved mechanisms to utilize chromosome breakage and end-healing to alter their genome structures during differentiation [6]. Previous studies have shown that programmed genome alterations occur in more than 100 species of diverse organisms including ciliates and vertebrates [7, 8]. Programmed genome alterations are co-related with gene silencing, dosage compensation and/or sex determination in these organisms [8].

Programmed DNA elimination is a prominent form of genome alteration, and was first discovered as chromatin diminution in the nematode *Parascaris univalens* [8, 9]. In *Ascaris suum* and *P*. *univalens*, 13% and 88% of genomic DNA, respectively, is eliminated during somatic cell differentiation [10–13]. This process removes all detectable heterochromatin from the somatic progenitor cells during early embryonic cleavages [8, 10]. In the pre-somatic cells of *A*. *suum*, telomere repeats are added *de novo* to all broken ends within a 4 to 6-kb region after chromosome breakage, including those destined for elimination [14, 15]. These fragments fail to attach to microtubules of the mitotic spindle in the anaphase, and are left in the cytoplasm and degraded after cell division [10, 12, 16]. Transcriptome analysis revealed that at least 685 germline expressed genes were eliminated from somatic cells [13], with only a few of them encode proteins with known functions [17–19].

**Fig 1:**
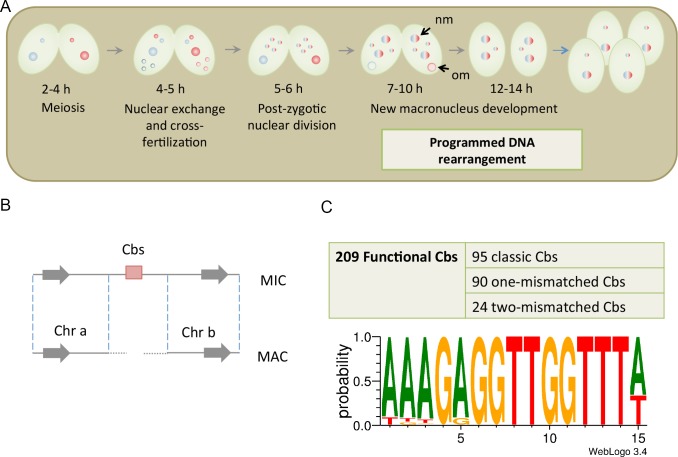
The characteristic of the chromosome breakage sequence (Cbs). (A) Sexual reproduction in *Tetrahymena*. (B) MAC formation is directed by Cbs breakage. After Cbs breakage, the MIC genome separates into independent MAC minichromosomes, and new telomeres are added to the ends of the MAC minichromosomes [39, 40]. (C) We identify 209 functional Cbs sites in the MIC genome. About half of them are classic Cbs with the exact same sequence [39]. The PWM (Position Weight Matrix) shows that there is an invariable 10-bp core sequence and the other 5 positions have the highest probability to be an "A", and limited possibilities for other nucleotide substitutions [42]. (nm: new macronucleus; om: old macronucleus)

Programmed DNA elimination has also been found in ciliated protozoa, including *Tetrahymena*, *Paramecium* [20, 21], *Stylonychia* [22–24], *Euplotes* [25] and *Oxytricha* [26]. *Tetrahymena thermophila*, like all ciliates, displays nuclear dualism and contains a somatic nucleus (macronucleus, MAC) and a germline nucleus (micronucleus, MIC) in the same cell. During the growth phase, the MAC undergoes amitotic division and the MIC divides by typical mitosis. During conjugation, the MIC goes through meiosis, mitosis and cross-fertilization to generate zygotic nuclei, which further divide and develop into new MAC and MIC. The developing new MAC undergoes a series of dramatic programmed DNA rearrangements, including the elimination of ~34% of the genome (from 157 Mb to 104 Mb) and the fragmentation of the 5 MIC chromosomes into about 225 minichromosomes that are retained in the MAC (Fig 1A) [27, 28]. Two globally occurring processes have been found: IES (internal elimination sequence) deletion and chromosome breakage. In IES deletion, defined DNA sections are deleted by a complex mechanism involving RNA-guided heterochromatin modification, boundary recognitions and DNA excision by a domesticated transposase [29–35]. The flanking DNAs are rejoined through a nonhomologous end-joining (NHEJ) pathway [36]. In chromosome breakage, approximately 200 specific sites are broken and telomeres are added to both ends after limited nucleotide loss [37–40], which produce chromosome fragments (minichromosomes) averaging 462 kb in size [41].

The molecular structure of a chromosome breakage site was first characterized at the rDNA (ribosomal RNA gene) locus of *Tetrahymena*. The rDNA exists as a single copy gene in the MIC genome [43]. During new MAC development, it is amplified to become ~9,000 copies of linear minichromosomes, each containing two copies of the gene as a head-to-head dimer [44–47]. Further studies revealed that specific chromosome breakage occurs at both ends of the rDNA, which share a 20-nucleotide sequence motif [47]. By searching for additional copies of similar sequences in the genome, more breakage sites were discovered and shown to contain the Chromosome breakage sequence (Cbs), a 15-bp element, AAAGAGGTTGGTTTA, which was later shown to be necessary and sufficient for chromosome breakage to occur [37, 39]. The breakage is coupled with *de novo* telomere addition within 30 bp of the Cbs [40]. Moreover, the sequences of Cbs are very well conserved. The copies of Cbs at the rDNA locus are nearly identical among six additional *Tetrahymena* and two related species. This finding suggested that Cbs also served as the chromosome breakage signal in these species [48]. Subsequently, Hamilton et al. (2006) examined 40 additional Cbs sites in *T*. *thermophila* and found that a 10-bp core of this 15-bp sequence was completely conserved. The other positions showed restricted one or two nucleotides variations [49].

Although chromosome breakage has been well studied in *Tetrahymena*, its genome-wide distribution and the overall fates of the minichromosomes generated remain unknown. One study has shown that a single MAC minichromosome end that was retained at or before 30 fissions after conjugation was lost after 100 fissions. This age-related loss could suggest the loss of the entire minichromosome and hinted at the loss of additional minichromosomes during ageing [50]. However, whether it plays a significant role in programmed DNA elimination, as it does in nematodes, is unclear. Here, we examined the issue of chromosome breakage and minichromosome formation at the genomic level in *Tetrahymena*. We analyzed all Cbs-related sequences in the MIC genome and correlate the potential breakage sites with the minichromosomes found in the MAC. Surprisingly, some of the predicted minichromosomes were absent among the MAC minichromosomes, revealing a new form of DNA elimination in *Tetrahymena*. We found that they are maintained through conjugation and are lost about 10 doublings after growth. Moreover, some of them encoded genes with possible functions in late conjugation. This finding uncovers a new layer of programmed DNA elimination in ciliates and offers a possible developmental gene regulation process through DNA elimination.

## Results

### Chromosome Breakage is Controlled by a Strong and Highly Conserved Motif

To assess the global distribution of chromosome breakage sites, we examined the publically available sequence databases for the MAC and MIC genome [41, 51, 52]. We searched MIC supercontigs for shared sequences at potential breakage sites, focusing first on the 10-kb intervals immediately adjacent to such sites, which we defined as sequences that correspond to MAC sequences adjacent to acquired new telomeres. We looked for motifs with significant similarity to the 15-bp classic Cbs or any other shared motifs. No other shared motifs were uncovered besides the Cbs-like sequences. A position frequency matrix (PFM) was generated by using these Cbs-like sequences. We then scanned the entire MIC genome for any additional Cbs-like sequence by using the PFM.

Any copy of Cbs-like sequence that separated one MIC chromosome into two corresponding MAC minichromosomes is regarded as a functional Cbs (Fig 1B). Although sometimes only one end can be found in the existing MAC genome database, 209 copies of functional Cbs are identified, which potentially could generate 208 minichromosomes in the MAC, accounting for over 90% of the 225 MAC minichromosomes previously identified from the MAC genome sequencing data [41]. Of these, 95 were identical to the classic Cbs sequence identified previously [39], 90 had one substitution and 24 had two substitutions. Noted that we found 10 additional copies of classic Cbs sequence inside of the MIC-specific regions. One of them was shown to be in the IES region, while others would be verified in the following analysis. All of these 15-bp sequences shared an identical 10-bp core and at most 2 substitutions in the remaining 5 positions, which agreed with previous findings based on a data set of 40 copies of Cbs [49]. Interestingly, these 5 variable positions are all composed of “A” in the classic Cbs. In position 1 and 15, they can only be substituted into “T”; in position 5, it can only be substituted into “G” (Fig 1C and S1 Table). We now define the term “Cbs” for the group of 15 bp sequences with these clear features, and the term “classic Cbs” for the exact 15 bp sequence originally described. To further confirm these breakage sites and determine any possible strain variations, we sequenced the MAC genome of three inbred lines using the Illumina paired-end sequencing method. We focused on the 118 Cbs sites for which both expected minichromosome ends could be identified in either the public database or our sequences of the three inbred lines. In all lines, breakages were found at all sites, strongly supporting the consistency of the breakage events. They all led to the loss of the 15-bp Cbs and small amounts of adjacent sequences, producing a gap averaging 56.79 bp (with 8.84 bp variations) before telomeres were acquired. Thus, Cbs appears to direct a well-regulated and consistent chromosome breakage process in *Tetrahymena*.

Since every MAC minichromosome end we have identified is derived from a MIC regions with a copy of Cbs, there appears to be no other sequence signal for chromosome breakage, and since all identified Cbs copies correspond to telomerized MAC DNA ends (where sequences are available), Cbs appears to be an obligatory breakage signal in this species.

### Chromosome Loss after Breakage at Cbs

To our surprise, several sections bounded by two Cbs sites are totally devoid of MAC reads when we mapped the MAC DNA reads into the MIC genome, suggesting their elimination from the mature somatic genome. They are not like IES, which are flanked by sequences that are joined back together in the MAC. In most of these cases a telomerized MAC DNA sequence could still be found within 30bp of the other side of these Cbs copies, thus the Cbs in these sites apparently are still functional to produce breaks, but the entire Cbs-bounded section is lost afterward.

**Fig 2:**
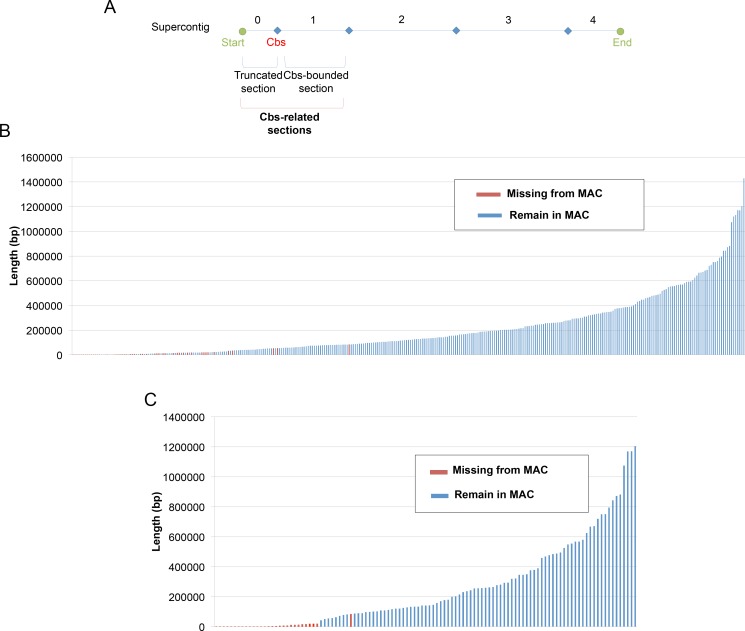
The size distribution of putative MAC minichromosomes. (A) We name the putative MAC minichromosome by its order in the MAC supercontig as illustrated. The section between the start position to the first Cbs site is named echr2.X.0, the following section between two Cbs sties is echr2.X.1 and so on. (B) The size distribution of all Cbs-related sections are shown. There are two kinds of Cbs-related sections. The red bar indicates the Cbs-related section that is eliminated from the MAC genome. The blue bar indicates the Cbs-related section that remains in the mature MAC genome. (C) The size distribution of only the Cbs-bounded sections are shown.

To further understand this phenomenon, we investigated the correlationship between Cbs sites and MAC minichromosomes. We found that the 218 Cbs sites detected were located in 107 MIC supercontigs (out of the total of 1,464 supercontigs) (S1 Fig), and defined 326 sections that were bounded either by Cbs on both sides (113 sections) or by Cbs and an end of a supercontig (213 sections) (Fig 2A). This is mostly due to the incompleteness of the MIC genome database. These sections are referred to as Cbs-related sections, which represent 69.66 Mb in total length, or 44.37% of the MIC genome. The other half (55.63%) of the MIC sequence is without a copy of Cbs and mostly in small supercontigs (S1 Fig). Among these Cbs-related sections, 276 are present in and 50 are eliminated from all mature MAC genomes analyzed (Fig 2B and S2 Table). The eliminated part is about 0.56 Mb, or 0.82%, of all Cbs-related sections (0.36% of the MIC genome). Among these sections, 113 are bounded by Cbs on both ends, totaling 29.29 Mb in length (42.05% of the entire Cbs-related sections and 18.66% of the MIC genome). Of these, 82 are present in, and 31 are eliminated from, the mature MAC (Fig 2C). Thus, in *Tetrahymena*, many minichromosomes are generated and lost during macronuclear formation, which reveals a new mode of DNA elimination in this species.

We then matched the Cbs-related sections to the corresponding MAC scaffolds with telomeres in both ends, which are full-length MAC minichromosomes. There are 129 full-length MAC minichromosomes in the public MAC genome database. We identified 184 Cbs-related sections with sequences matching all or a part of these 129 full-length MAC minichromosomes. 67 of these sections are bounded in both ends by Cbs and can be used for direct one-to-one comparison with MAC minichromosomes. We found that MAC minichromosomes are similar or slightly shorter, but never longer, than the corresponding Cbs-bounded sections (S2 Fig). It suggests that MAC minichromosomes are derived from Cbs-bounded sections through chromosome breakage without complicated rearrangements such as interchromosomal translocation. The reductions in size are mostly the outcomes of IES deletions.

In general, we observed that the eliminated sections (30 bp to 83.8 kb) are shorter than those retained in the mature MAC (42.31 kb to 1.43 Mb). To see whether the eliminated sections are clustered in the genome, we mapped them onto the MIC supercontigs (S3 Fig) and found that they are not clustered in general. However, some of them are located at continuous regions, including the 9 Cbs sites that we found in the MIC-specific regions. The two smallest 30-bp eliminated sections (echr2.2.1 and echr2.2.2) are both located near the rRNA gene. It has been shown previously that the sequences between these two sections are nearly identical (S4 Fig) [39]. We also identified another 4 eliminated sections (echr2.264.1 to echr2.264.4) that are present as direct tandem repeats. This region includes 5 copies of Cbs plus one copy of a similar sequence, each as a part of a 120-bp repeating unit that share more than 90% similarity among the 6 copies (S4 Fig). Although the majority of the eliminated sections are distributed separately, this result suggests that some small-sized eliminated sections might have arisen from sequence duplication. Our observations indicate that the eliminated sections could be generated by Cbs breakage through a mechanism of DNA elimination that is distinct from IES deletion in *Tetrahymena thermophila*.

### Eliminated Minichromosomes Disappeared after Conjugation

**Fig 3:**
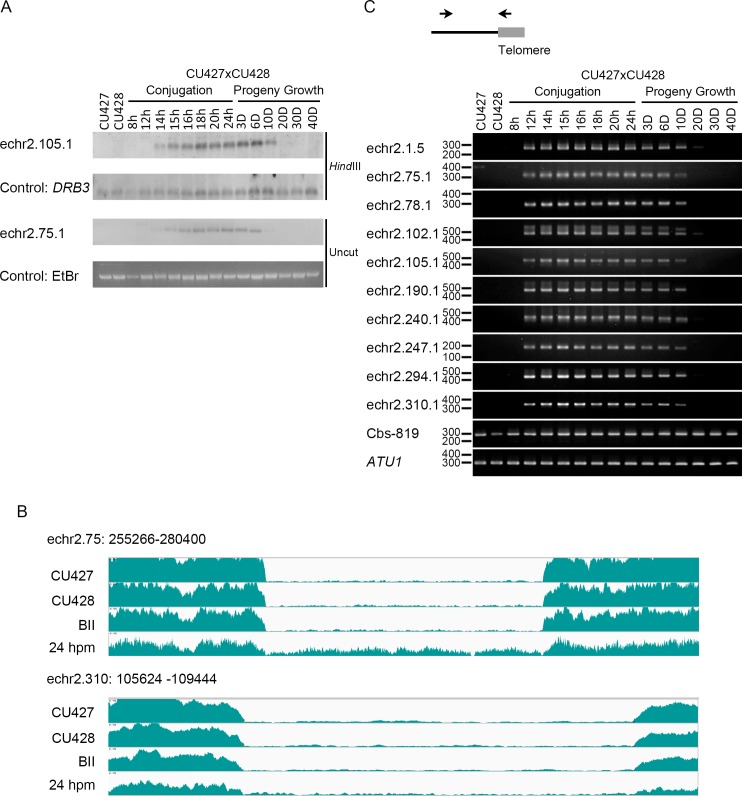
Eliminated minichromosomes disappear at different time points. (A) Southern blot hybridization of echr2.105.1 and echr2.75.1 are shown. The genomic DNA extracted from vegetative cells, conjugating cells (8, 12, 14, 15, 16, 18, 20 and 24 hour post mixing) and feeding progeny cells (3, 6, 10, 20, 30 and 40 doublings after conjugation) was analyzed. *Hind*III digested (for echr2.105.1) and uncut whole cell DNA samples (for echr2.75.1) were separated in a 0.6% agarose gel by standard gel electrophoresis, respectively. *DRB3* gene was used as a macronucleus-retained DNA control for *Hind*III-cut DNA, and ethidium bromide (EtBr) staining of uncut DNA was used for loading control. D: doubling number after conjugation. (B) The coverages of reads of DNA in the growing phase of the inbred lines CU427, CU428, BII and in the mating pool (24hpm) are shown. We use the MIC genome as the reference to align these whole cell DNA reads, which are mostly MAC reads. The region with low coverages in CU427, CU428 and BII indicates the location of the eliminated minichromosome. The coverage of the region in supercont2.75, but not in supercont2.310, is slightly higher in the mating pool. It should be noted that the mating pool contains significant amounts (~30%) of non-mated cells and aborted mating cells that contain mature MAC, which partially explain the lower coverage in this section than its neighboring sections. (C) Telomere-anchored PCR of one end of 10 EMCs is shown. EMCs were detected by PCR using telomere sequence as one primer and the sequence of the minichromosome as the other (black arrows). The black line indicates one end of a minichromosome in the macronucleus, and the gray box indicates telomere. The genomic DNA of vegetative cells, different stages of conjugating cells and feeding progeny cells after conjugation was analyzed. The chromosome ends near Cbs819 and ATU1 (α-tubulin) were used as the macronucleus-retained DNA control.

To address the elimination process, we investigated the appearance of the eliminated sections at different time points during mating. We first focused on the largest (83-kb) eliminated minichromosome (EMC), echr2.105.1, by Southern blot hybridization to detect one of the terminal fragments with the predicted telomere, which first appeared at the stage of 14 hours post mixing (hpm) of starved cells to initiate mating, matching the known timing of Cbs breakage. Surprisingly, we found that it was maintained throughout the mating process (Fig 3A and S5 Fig). Another eliminated minichromosome, echr2.75.1, which is 12 kb long and can be separated from the bulk DNA in gel electrophoresis without restriction enzyme digestion, shows a similar result (Fig 3A). In addition, the DNA amounts seemed to have increased after Cbs breakage, and continued to be maintained at a level similar to the bulk of the MAC DNA even after feeding and growth for approximately 6 doublings. They became significantly reduced at 10 doublings and undetectable at 20 doublings (Fig 3A). The initial increase in DNA content is similar to the endoduplication of the MAC-destined minichromosomes, which duplicate successively without division to generate the estimated 67 copies found in the mature MAC. After conjugation, these minichromosomes appear to keep pace with the bulk DNA in relative abundance for 6 cell doublings before elimination occurs, indicating the ability to replicate until this stage. The loss could be caused by a failure in DNA replication and/or segregation or by active degradation.

To determine how many EMCs are lost only after growth, we sequenced MAC DNA of a mating pool at the 24 hpm stage. We found that most of the EMCs remained at this stage, but some appeared to have much lower read coverage (Fig 3B and S6 Fig). To determine whether elimination occurs before this stage for some minichromosomes, we developed a PCR assay to detect specific ends with telomeres produced after breakages. When we examined 10 EMCs (including the two detected by Southern hybridization), we were surprised to find that, regardless of their read coverage, every tested EMC showed a similar pattern (Fig 3C), appearing at 12 hpm, maintained through 6 doublings of growth and eliminated before 20 doublings. Comparing the Southern hybridization and the PCR results, we concluded that the reduction occurred largely by 10 doublings, but some amounts still remained and were detectable later due to the higher sensitivity of PCR. Although some minichromosomes not analyzed may still be eliminated at other time points, there appears to be a major developmental window for their occurrence, which is at about 10 doublings. This implies a regulation in DNA replication/segregation or degradation at this stage. The differences in sequence coverage we have observed in the 24 hpm sample are not due to elimination time differences, and could be the results of differences in IES contents as described below.

To determine whether EMCs are specifically associated with heterochromatin, in a way similar to heterochromatin association for IES elimination, we analyzed the publically available ChIP-seq analysis using the anti-Pdd1p antibody obtained from cells at the 12 hpm stage. Pdd1p, a HP-like protein, is known to be associated with the heterochromatinized IESs and plays an essential role in IES elimination [53–55]. We found that the average fold enrichment of Pdd1p is high in both IESs (0.68) and EMCs (0.60), which is 2.9 folds and 2.6 folds higher than in all genomic sequences present in contigs with Cbs (the Cbs-related sections) that contain both MAC-destined Sequences (MDSs) and IESs (0.23) (S7 Fig). However, the distribution is uneven, and in 27 of these 50 EMCs it is lower than 0.23 (S3 Table). We wondered if IES were present in some EMCs, which could not be determined using existing genomic data. We thus directly examined several EMCs for the presence of IES by PCR and sequencing. We were surprised to find exceptionally large amounts of IES (a 8.9-kb IES in the 10-kb echr2.78.1 and a 2.8-kb IES in the 3.8-kb echr2.310.1, S8 Fig, which reduced the two minichromosomes to about 1.4 kb and 1 kb, respectively). Moreover, these IES regions coincide with the Pdd1p occupied regions. The result implied that the high amount of Pdd1p association could be due to IES elimination and probably not EMC markings, and at least partially explained the low read coverage of these EMCs at 24 hpm.

### Genes in Eliminated Minichromosomes are Expressed during Conjugation

**Fig 4:**
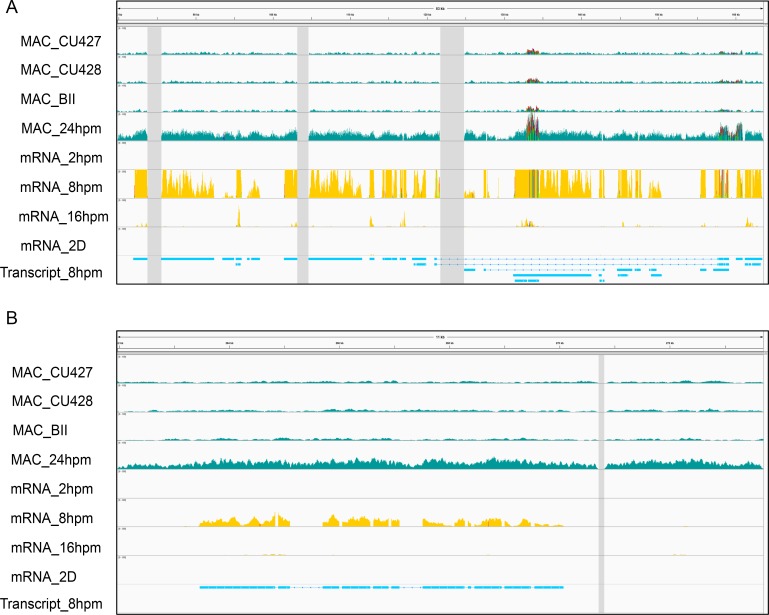
Eliminated minichromosomes express genes in the late conjugation stage. This figure shows the read coverage of genomic DNA and mRNA at different time points in echr2.105.1 (A) and in echr2.75.1 (B). MAC: MAC genomic sequencing results. Expression: mRNA sequencing results. hpm: hours post mixing. D: doubling number after conjugation. Transcript: the predicted transcript from Cufflinks [57]. Gray boxes indicate the unknown region caused by sequencing incompletion.

The eliminated minichromsomes have a brief existence in the new MAC and thus have potentials to play stage-specific roles. We explored this possibility by analyzing their transcripts. We examined the transcriptomes of growing cells and cells at the 2 hpm and the 8 hpm stages in the public sequencing database [56], as well as generated RNA sequence data for cells at the 16 hpm stage and at 2 doublings after conjugation. These transcripts could be detected at the 8 hpm stage and most of them disappeared at the time of 2 doublings after conjugation (Table 1 and Fig 4). None of them were expressed during growth or at the 2 hpm stage as expected for sequences not present in the MAC. Only 10 of the EMCs expressed transcripts, and at least 43 genes were identified (Table 1). This gives one gene for every 11.67 kb of DNA in these eliminated minichromosomes (with the total length of 0.50 Mb), which is about three folds less than the overall gene density in the MAC (one gene for every 4.17 kb). For comparison, we found 239 genes that were expressed at these time points from the 9,479 IESs we had tentatively identified in the MIC genome. The total length of these IESs is 44.8 Mb, giving rise to one gene in every 188 kb of IESs. The gene density of the eliminated chromosomes is thus much higher than that of the IES regions.

**Table 1:** The number of expressed genes in eliminated minichromosomes.

	Chromosome ID	Length(bp)	2h expressed gene	8h expressed gene	16h expressed gene	2D expressed gene
Cbs-bounded	echr2.105.1	83,803	0	21	4	3
	echr2.75.1	11,738	0	1	1	0
	echr2.221.1–5 (cluster)	68,129	0	15	14	1
	echr2.273.2	15,837	0	0	1	1
Cbs-related	echr2.190.1	52,188	0	3	4	1
	echr2.800.0	12,070	0	1	1	0

All expressed genes are presented according to stages of expression. echr2.221 indicates a region that contains the cluster of eliminated minichromosomes from echr2.221.1 to echr2.221.5. D: doubling number after conjugation

**Fig 5:**
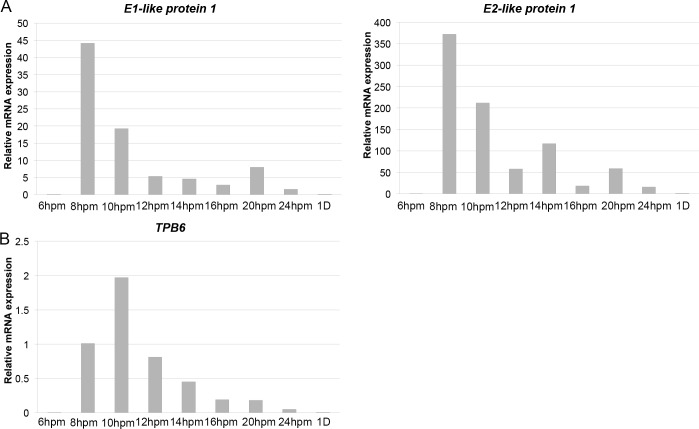
The expression profile of genes in the eliminated minichromosomes. (A) shows the quantitative-RT-PCR results of two genes located in echr2.105.1. (B) shows the quantitative-RT-PCR result of the gene located in echr2.75.1. hpm: hours post mixing. D: doubling number after conjugation.

To further investigate their possible functions, we analyzed the sequence for encoded proteins. Most of the predicted proteins do not have a conserved domain. However, we found 5 participating in ubiquitin signaling pathways in echr2.105.1 (Fig 4A), with two encoding the ubiquitin-activating enzyme E1 (S9 Fig) and three encoding the ubiquitin-conjugating enzyme E2 (S10 Fig). The *E1-like protein 1* and *E2-like protein 1* show the highest transcription levels at the 8 hpm stage, which is earlier than the time of Cbs breakage (Fig 5A). Their expression profiles are very similar, implying related functions. In addition, we found a gene in echr2.75.1 that encoded a paralog of the domesticated *piggyBac* transposase *TPB1* (Fig 4B), and named it *TPB6*. The peak of expression of *TPB6* is at the 10 hpm stage (Fig 5B), which is similar to that of *TPB1*. The results suggest that genes in the eliminated chromosomes could play roles in protein regulations and DNA rearrangements.

### Some Eliminated Minichromosomes are Retained in the MAC in Other *Tetrahymena* Species

**Fig 6:**
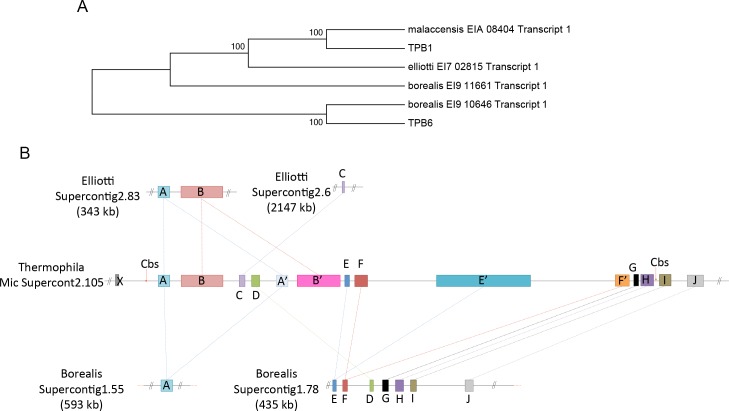
Evolution of eliminated minichromosomes. (A) The phylogenetic tree shows that *TPB6* only has one ortholog in *T*. *borealis*. (B) Genes in echr2.105.1 in *T*. *thermophila* are conserved in other *Tetrahymena* species. Gene X, I and J are MAC-destined genes in both *T*. *thermophila* and *T*. *borealis*. The figure indicates the synteny of the genes in the 3’-truncated region of echr2.105.1 and the adjacent MAC minichromosome in these species.

Because the EMCs carry genes with possible functions during late conjugation, it would be interesting to find out if this process is conserved in other *Tetrahymena* species. We first searched for these genes in the available MAC genome databases of three other *Tetrahymena* species: *T*. *malaccensis*, *T*. *elliotti*, and *T*. *borealis* [58]. We could not find any orthologues of these genes in the closest species, *T*. *malaccensis*, agreeing with the expectation that these regions were also eliminated from the MAC of *T*. *malaccensis*. However, several of them were found in the more distantly related *T*. *elliotti* and *T*. *borealis* MAC genome (S4 Table), suggesting that they were not eliminated (Fig 6).

*TPB6* offered some insights in this regard. We found a highly conserved ortholog in the MAC genome of *T*. *borealis*, the most distant of the three species from *T*. *thermophila*. Phylogenic analysis suggests that these two *TPB6* genes are more closely related to each other than to *TPB1*, a paralog of *TPB6* found in the MAC genome of all four *Tetrahymena* species analyzed (Fig 6A), and shared both the conserved Ku and DDD domains (S11 Fig). However, we cannot find synteny between these two species in the upstream or downstream genes of echr2.75.1, suggesting that *TPB6* may be located in a different region in the *T*. *borealis* MIC genome. On the other hand, several genes in other eliminated chromosomes show syntenies between the *T*. *elliotti* and *T*. *borealis* MAC genome (Fig 6B). For instance, we found orthologous for most genes except gene B and C in echr2.105.1 in the *T*. *borealis* MAC genome. Although the gene order is slightly different, the 3’-truncated part of echr2.105.1 in *T*. *borealis* is links to the adjacent MAC minichromosome in the same configuration as in the *T*. *thermophila* MIC genome. The Cbs presents in the corresponding gap (between gene H and I, Fig 6B) in *T*. *thermophila* is absent from *T*. *borealis*, suggesting either a Cbs site generated in *T*. *thermophila* or lost from *T*. *borealis*. The result shows that the MIC-specific gene could be a MAC-destined gene in other *Tetrahymena* species.

## Discussion

This study reveals a new mode of programmed DNA elimination in *Tetrahymena* through chromosome breakage. Previous understanding of DNA elimination in *T*. *thermophila* has been limited to internal DNA deletion. However, our results indicate that the breakage at Cbs site causes not only chromosome fragmentation but also somatic genome reduction. Thus, similar to nematodes, chromosome breakages also lead to programmed DNA elimination in *Tetrahymena*.

Analysis of the MAC and MIC genome revealed the sequence nature and fates of genomic Cbs copies during nuclear differentiation. We found that these copies are highly similar, either with an identical 15-bp sequence or with a maximum of two nucleotide substitutions restricted to 5 of the positions. All breakages led to the loss of Cbs plus the immediate flanking sequences. The gaps created are small and similar in sizes (57.79 bp in average; s.d. = 8.84), which are much smaller than those in *Paramecium tetraurelia* (200–500 bp) [21] or in *A*. *suum* (4–6 kb) [11]. Furthermore, the lost sequences in either side of the Cbs show a significant 5-bp size difference (p-value = 3.85e-31), being 18.8 bp (s.d. = 5.3) in the 5’- and 23.9 bp (s.d. = 6.9) in the 3’-flanking region of the G strand of Cbs. Interestingly, if one measures the distances to the conserved 10 nucleotides core (from position 6^th^ to 14^th^) instead of the 15 bp Cbs, then the difference disappears, further suggesting the importance of this core sequence in cutting site selection. These features together suggest a well-regulated molecular event that couples double-stranded DNA breaks at Cbs and limited DNA loss with new telomere acquisition.

We found that the minichromosomes generated by breakages at Cbs had two possible fates: maintained throughout vegetative growth to constitute the mature MAC genome, or maintained only during conjugation, and are absent in the mature MAC. Both classes appeared to be endoduplicated during conjugation, steadily increased their amounts until the end of this period. The first class was maintained whereas the second class disappeared at certain point after cells resumed growth and division. The second class should be able to replicate during the initial doublings, otherwise they would be about 64-fold reduced at 6 doublings after conjugation, which was not observed. Their disappearance beginning at around 10 doublings after conjugation suggests the onset of a regulatory process in differential DNA replication/segregation or degradation. The mechanism of this elimination process would be interesting to discern. It is possible that these EMCs, many smaller than 10 kb, have replication origins that become dysfunction at this time through changes in chromatin structure or changes in specific regulatory protein availability. The disability leads these EMCs to stop replicating and be diluted out by cell divisions. Another possibility is the active degradation of these EMCs at this time, which implies a sequence-specific DNA degradation machinery to regulate this process. Either possibility implied programmed changes in cellular activities during early vegetative growth. It is interesting to note that after conjugation, *Tetrahymena* reaches sexual maturity only after about 40 doublings. Thus, the elimination of EMCs occurs during the time when the new progeny is still maturing, presumably accompanied by programmed changes in cellular activities. Furthermore, like the first class, the second class also contained genes, but their expressions were limited to late conjugation stages, possibly contribute to new MAC development and other stage-specific processes. Since elimination occurs later than the expression period, it likely plays little role in their down regulation. Nonetheless, the process ensures that no re-expression of these genes can occur during later growth. It is interesting to note that programmed DNA elimination, through a yet unknown mechanism, may regulate specific telomerase gene expression in the distantly related ciliate *Euplotes* [59].

The largest eliminated minichromosome we have identified is 83 kb in size, shorter than the average of the retained chromosomes. However, due to the incompleteness of the MIC genome sequence data, there could still be longer ones. For instance, there are 6 truncated sections of eliminated minichromsomes with only one end defined (the other end is the MIC supercontig end) that are 20 kb to 55 kb in length (S2 Table). Thus the entire eliminated minichromosomes should be bigger, possibly even bigger than 83 kb. Overall, we are able to assign eliminated minichromosomes to only the 44.54% of the sequenced MIC genome that are covered by supercontigs with at least one Cbs, of which they represent 0.82% (or 570 kb). This accounts for a relatively small proportion of the ~34% of the genome that is eliminated, most of which presumably are IESs. However, we cannot rule out the possibility that a higher proportion of the remaining 55.46% of the genome is eliminated through chromosome breakage. Two observations support this possibility. Firstly, in the S2 Fig, we determined the difference in length between the MAC minichromosomes and the corresponding Cbs-bounded sections. It offers an assessment of the amount of genome reduction caused solely by IES eliminations and not by minichromosome elimination. We found only 2.96 Mb of IES in the 67 Cbs-bounded sections (24.35 Mb in total, 15% of the MIC genome), which represented only 12% of this part of the genome and thus is ~22% lower than the expected ~34%. If this figure is representative of the rest of the genome, there would be a lot of room left for mechanisms other than IES elimination, and minichromosome elimination can be a major factor. Secondly, when we determined the amount of IES using the program BreakDancer, which could detect internal deletions in the MAC genome when compared with the MIC genome, we identified 9,479 IESs totaling 44.8 Mb. This is significantly smaller than the 51.1 Mb known to be eliminated, leaving room for additional loss of the somatic genome by minichromosome elimination. Thus we propose that minichromosome elimination can play a major role in re-structuring the somatic genome of *Tetrahymena*, not only in altering its integrity but also in reducing its content, and possibly include the elimination of centromeres and MIC-specific telomeres, the mechanism for their loss in ciliates is still unknown.

## Material and Methods

### Cell and cell culture

Inbreeding *T*. *thermophila* strains B2086 II, CU427 (Chx/Chx [VI, cy-s]), and CU428 (Mpr/Mpr [VII, mp-s]) were obtained from Peter Bruns (Cornell University, Ithaca, NY). The method for maintaining and growing cells was as described by Gorovsky et al. (1975) [60]. *Tetrahymena* cells were grown in NEFF medium (0.25% proteose peptone [BD, New Jersey, USA], 0.25% yeast extract [BD], 0.5% dextrose [AMRESCO LLC, OH, USA], 0.022% Ferric Chloride [Sigma-Aldrich Corp., St. Louis, MO, USA]) at 30°C. Cells were prepared for mating by washing with 10 mM Tris-HCl (pH 7.4) buffer and incubating overnight before mixing. After 24 hours of mating, the cell mixture was transferred into NEFF medium for re-feeding. Drug selection against non-progeny cells was done by adding cycloheximide at one doubling after conjugation. Cell doublings were estimated by measuring cell densities.

### Genomic DNA sequencing and alignment

Genomic DNA was prepared using methods previously described [61]. We sequenced the library of WT strains to a depth of 49–60 million read-pairs with 2x100 bp using illumina HiSeq 2000 paired-end sequencing (Illumina Inc., San Diego, CA, USA) at the BRC NGS Core Facility in Academia Sinica (Taiwan) and the library of WT mating pool to a depth of 73 million read-pairs with 2x49 bp using Illumina HiSeq 2500 paired-end sequencing at the Fred Hutch Cancer Research Center (USA). The raw sequence data sets have been deposited at NCBI BioProject (http://www.ncbi.nlm.nih.gov/bioproject) as PRJNA326452. Sequencing quality was measured using FastQC software (http://www.bioinformatics.babraham.ac.uk/projects/fastqc). Quality scores across all bases were confirmed to be more than 30. Sequence alignment was mapped into the MIC genome assembly data [58] as the reference genome using bowtie2 [62] and SAM/BAM file handling was done by SAMtools [63]. The mapped reads were visualized using the Integrative Genomics Viewer (IGV) [64] and analyzed using custom Perl scripts.

### Southern blotting

To examine the eliminated minichromosomes, genomic DNA was extracted from vegetative cells, conjugating and re-fed cells at different time points. For standard gel electrophoresis, total DNAs were extracted from cells at different time points as previously described [61]. Uncut or *Hind*III-digested DNA were separated in a 0.6% agarose gel. After electrophoresis, DNA was transferred to a piece of IMMOBILON-NY+ nylon membrane (Millipore, Bedford, MA). The digoxigenin-labeled probes were generated using DIG High Prime DNA Labeling and Detection Starter Kit II (Roche, Indianapolis, IN) and mixed with the membrane for hybridization at 42°C overnight. The membranes were washed in 2× saline-sodium citrate (SSC) with 0.1% SDS twice and 0.5× SSC with 0.1% SDS at 65°C twice before detection of the luminescence using x-ray films.

### Telomere-anchored PCR

Genomic DNA was purified from vegetative cells, conjugating and re-fed cells collected at different time points. One end of 10 eliminated minichromosomes were examined by PCR analysis using a telomeric sequence as one of the two primers. All the PCR products were confirmed by sequencing. The primers used in the experiment are listed (S5 Table).

### EMC analysis

The sequence coverage at each position was normalized by the genome coverage in the MAC regions, which is 46x in the 24-hpm sample and 119x in the CU427 sample. At first, we calculated the differences of EMCs between the 24-hpm sample and the CU427 sample. Second, the frequency of differences was computed using Perl and the distribution of frequency was plotted using R. Third, each EMC was divided into 100 partitions and the average of the difference in each partition was calculated using Perl, and was plotted using R.

### mRNA sequencing and alignment

Total RNA in the 16 hpm stage and the 2 doubling stage after conjugation were purified using TriPure Isolation Reagent (Roche, Indianapolis, IN) and the purified RNAs were further concentrated using RNeasy MinElute Cleanup (QIAGEN, Hilden, Germany). PolyA-selected RNAs were obtained using TruSeq Stranded mRNA LT Sample Prep Kit (illumina), fragmented, and transcribed into cDNA library using Invitrogen SuperScript Double-Stranded cDNA Synthesis. We sequenced the library to a depth of 20 million read-pairs with 2x75 bp using illumina MiSeq paired-end sequencing (Illumina Inc., San Diego, CA, USA) at the IMB Genomic Core in Academia Sinica (Taiwan). The raw sequence data sets have been deposited at the NCBI BioProject (http://www.ncbi.nlm.nih.gov/bioproject) as PRJNA326452. The public sequencing data of mRNA in the growth condition, the 2 hpm, and the 8 hpm were downloaded from GEO, of the accession number under GSE27971 including GSM692081, GSM692085, and GSM692086 [56]. Sequencing quality was measured using FastQC software (http://www.bioinformatics.babraham.ac.uk/projects/fastqc). Quality scores across all bases were confirmed to be more than 30. Sequence alignment was mapped into the MIC genome assembly data [52] as the reference genome using bowtie2 [62] and SAM/BAM file handling was done by SAMtools [63]. Transcripts are assembled using TopHat and Cufflinks [57]. The differentially expressed genes and transcripts were discovered using cuffmerge and cuffdiff software [57] and custom Perl scripts.

### RT-PCR

Total RNA samples from different stages of conjugating and feeding progeny cells (CU427×CU428) were prepared using a RNA isolation kit (Roche, Indianapolis, IN) and were synthesized into first strand cDNA using Transcriptor reverse transcriptase with anchored-oligo(dT)_18_ primer. Quantitative-PCR analysis was performed using LightCycler Carousel-Based PCR System with the LightCycler FastStart DNA Masterplus SYBR Green kit (Roche). α-tubulin mRNA expression was an internal control for quantitative normalization. The primers used in quantitative-PCR are listed in S5 Table.

### Phylogenetic analysis

Evolutionary analyses were inferred using the Neighbor-Joining method [65], which were conducted in MEGA5 [66]. The bootstrap test replicates 1000 times which represents the percentage of replicate trees are shown next to the branches [67]. The Poisson correction method was used to compute the evolutionary distances [68] that are in the units of the number of amino acid substitutions per site. A gamma distribution (shape parameter = 1) was used to model the rate variation among sites. We eliminated the positions that contain gaps and missing data.

### Accession numbers

The accession number for the sequencing data is BioProject: PRJNA326452.

## Supporting Information

S1 Fig The length distribution of MIC supercontigs. Each bar represents the length of MIC supercontigs. The MIC supercontigs with Cbs sites (blue bar) and without (green bar) are illustrated.
(TIF)

S2 Fig Length comparison between the Cbs-bounded sections and their corresponding MAC minichromosomes. Here we only consider the MAC scaffolds that contain telomere sequences at both ends. Only 67 Cbs-bounded sections match with those MAC minichromosomes (green diamond). Blue cross: eliminated minichromosome. Red dash line indicates the same value in both axes. Black line indicates the trend line between the Cbs-bounded sections and the MAC minichromosomes.
(TIF)

S3 Fig The arrangement of eliminated Cbs-bounded sections. Each line indicates a different MIC supercontig, and the numbering method is identical to that in Fig 2A. The red letter indicates the Cbs-bounded section that is eliminated from the MAC genome.
(TIF)

S4 Fig Tandem repeats in eliminated minichromosomes. (A) Two eliminated minichromosomes show tandem repeats that are bounded by Cbs sites. Black bold letters: Cbs; Red bold letters: degenerate Cbs. (B) Sequence comparison of the repeats shown in (A).
(TIF)

S5 Fig Southern blot hybridization of echr2.105.1. Uncut whole cell DNA samples were collected at different time points (hours after mixing cells for mating and hours or doubling after conjugation) and separated in an agarose gel by pulsed-field gel electrophoresis. The doubling time is about 3 hours in growing population.
(TIF)

S6 Fig Difference calculation between the 24-hpm and CU427 samples of EMCs. (A) Accumulated Frequency of DNA coverage differences. 56.5% of the counts show in the difference less than 0.1, indicating the region with low coverage. (B) and (C) Distribution of differences for EMCs. X-axis indicates the percentage of length. Each EMC is divided into 100 bins, and the average difference of each bin is shown.
(TIF)

S7 Fig The fold enrichment of Pdd1p-bounded region in the MIC genome. The amount of fold enrichment of MDS, Cbs-related section, EMC and IES is 0.04, 0.23, 0.60 and 0.68, respectively. MDS: MAC-destine sequence; Cbs-related: Cbs-relate section; EMC: eliminated minichromosome; IES: internal eliminated sequence.
(TIF)

S8 Fig The distribution of Pdd1p in two EMCs. The read coverage of gDNA and the Pdd1p-bound region at echr2.78.1 (A) and echr2.310.1 (B). The red boxes indicate the region of IES.
(TIF)

S9 Fig Sequence comparison of the conserved domain of E1 homologous in *T*. *thermophila* and *Saccharomyces cerevisiae*. *CUFF*.*4210*.*1* indicates the *E1-like protein 1* and *CUFF*.*4196*.*1* indicates the other E1-like gene identified in this study.
(TIF)

S10 Fig Sequence comparison of the conserved domain of E2 homologous in *T*. *thermophila* and *Saccharomyces cerevisiae*. *CUFF*.*4092*.*1* indicates the *E2-like protein 1*, *CUFF*.*4092*.*2* and *CUFF*.*4182*.*1* indicate other E2-like genes identified in this study.
(TIF)

S11 Fig Sequence comparison of Tpb6p and the ortholog in *T*. *borealis*. Blue box: the conserved Ku domain. Red box: the catalytic DDD domain of *piggyBac* transposase.
(TIF)

S1 Table The frequency of nucleotide substitutions in each position of Cbs. (DOCX)

S2 Table List of eliminated minichromosomes. (DOCX)

S3 Table The fold enrichment of Pdd1p in EMCs. (DOCX)

S4 Table The conserved genes in other *Tetrahymena* species. (DOCX)

S5 Table Primer List. (DOCX)

S1 Text Supplemental methods. (DOCX)
