# Supplementary figures and images for: Programmed Minichromosome Elimination as a Mechanism for Somatic Genome Reduction in Tetrahymena thermophila
Source: PLoS Genet. 2016 Nov 2;12(11):e1006403. doi: 10.1371/journal.pgen.1006403 (PMC5091840; doi:10.1371/journal.pgen.1006403)

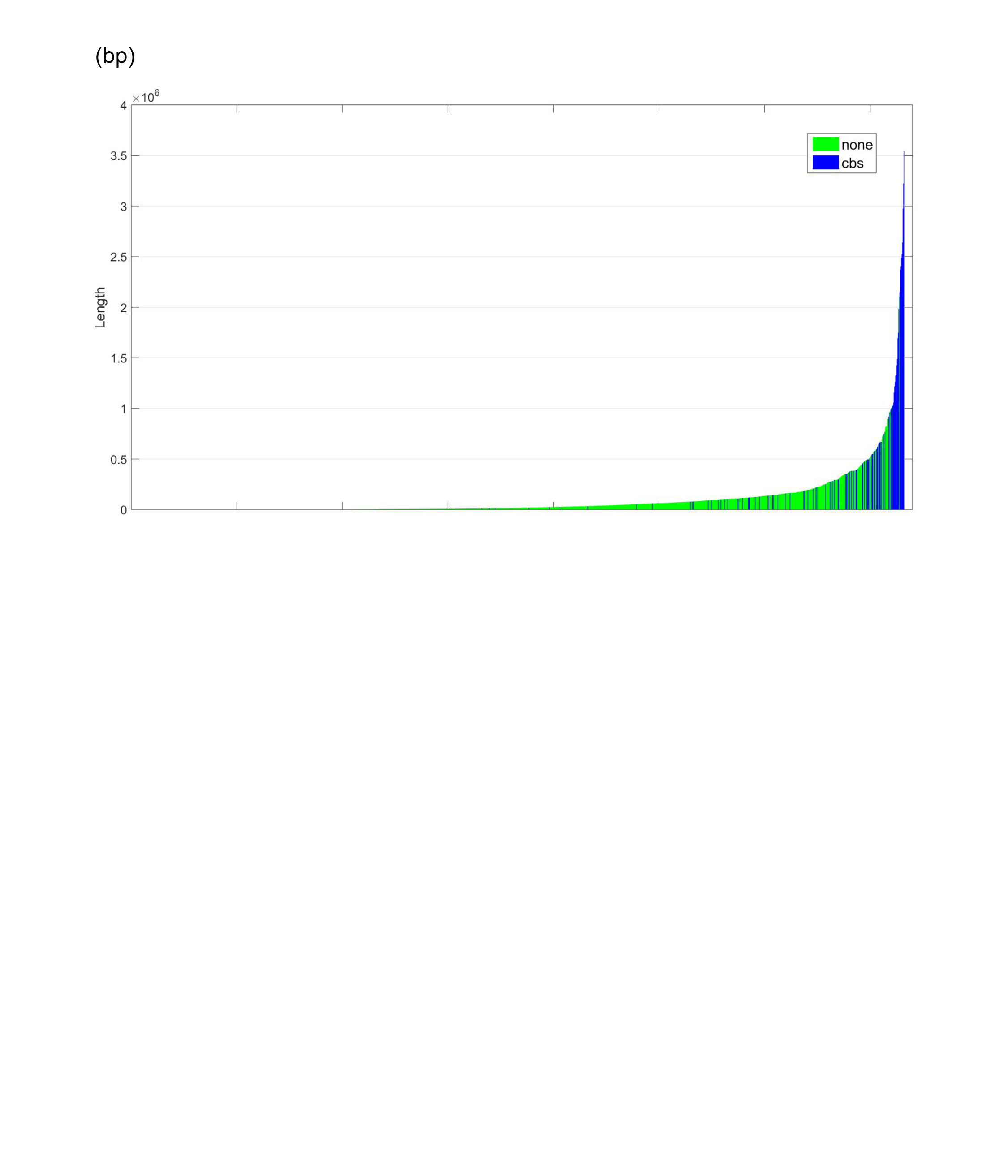

Supplement: S1 Fig — Each bar represents the length of MIC supercontigs. The MIC supercontigs with Cbs sites (blue bar) and without (green bar) are illustrated. (TIF) [file pgen.1006403.s001.tif]

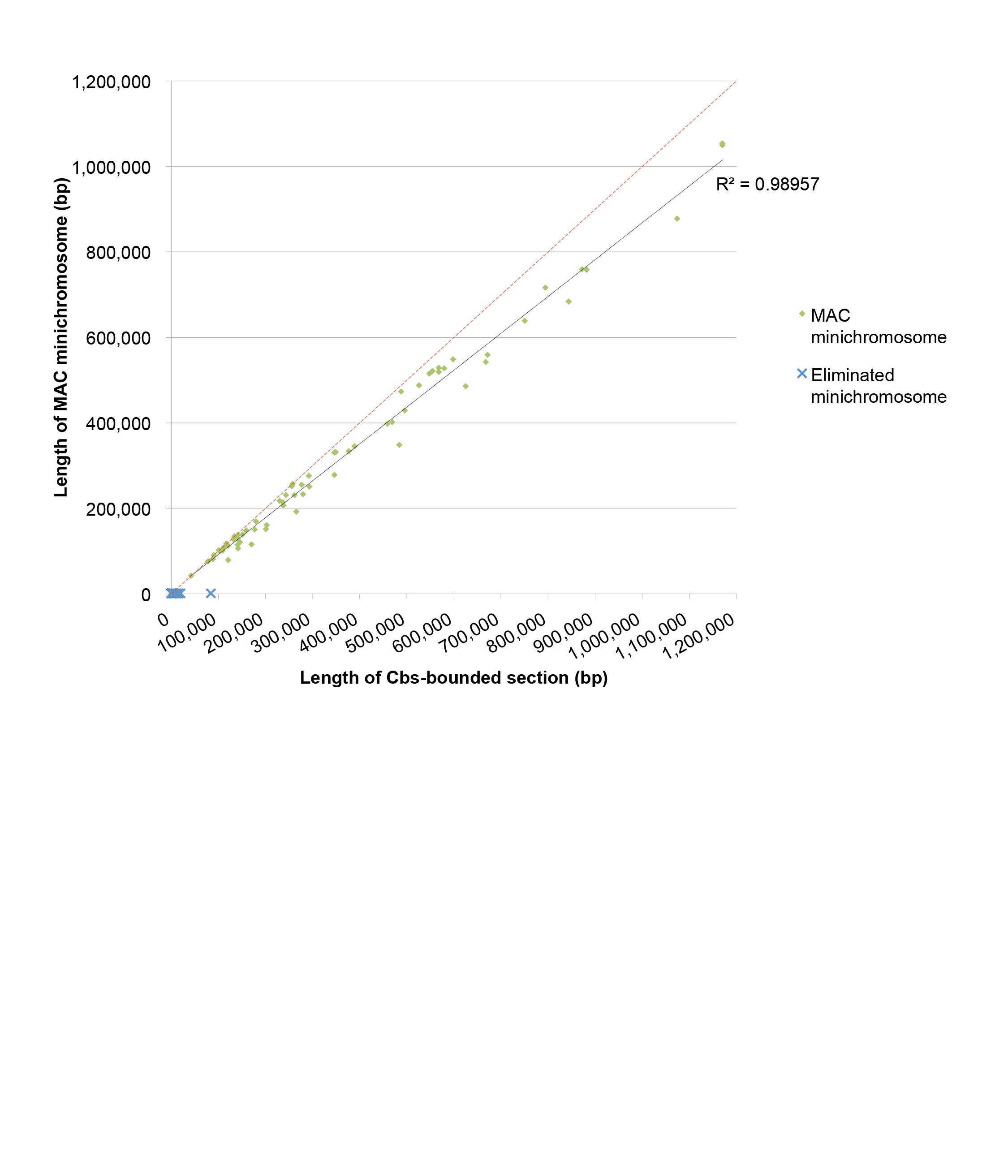

Supplement: S2 Fig — Here we only consider the MAC scaffolds that contain telomere sequences at both ends. Only 67 Cbs-bounded sections match with those MAC minichromosomes (green diamond). Blue cross: eliminated minichromosome. Red dash line indicates the same value in both axes. Black line indicates the trend line between the Cbs-bounded sections and the MAC minichromosomes. (TIF) [file pgen.1006403.s002.tif]

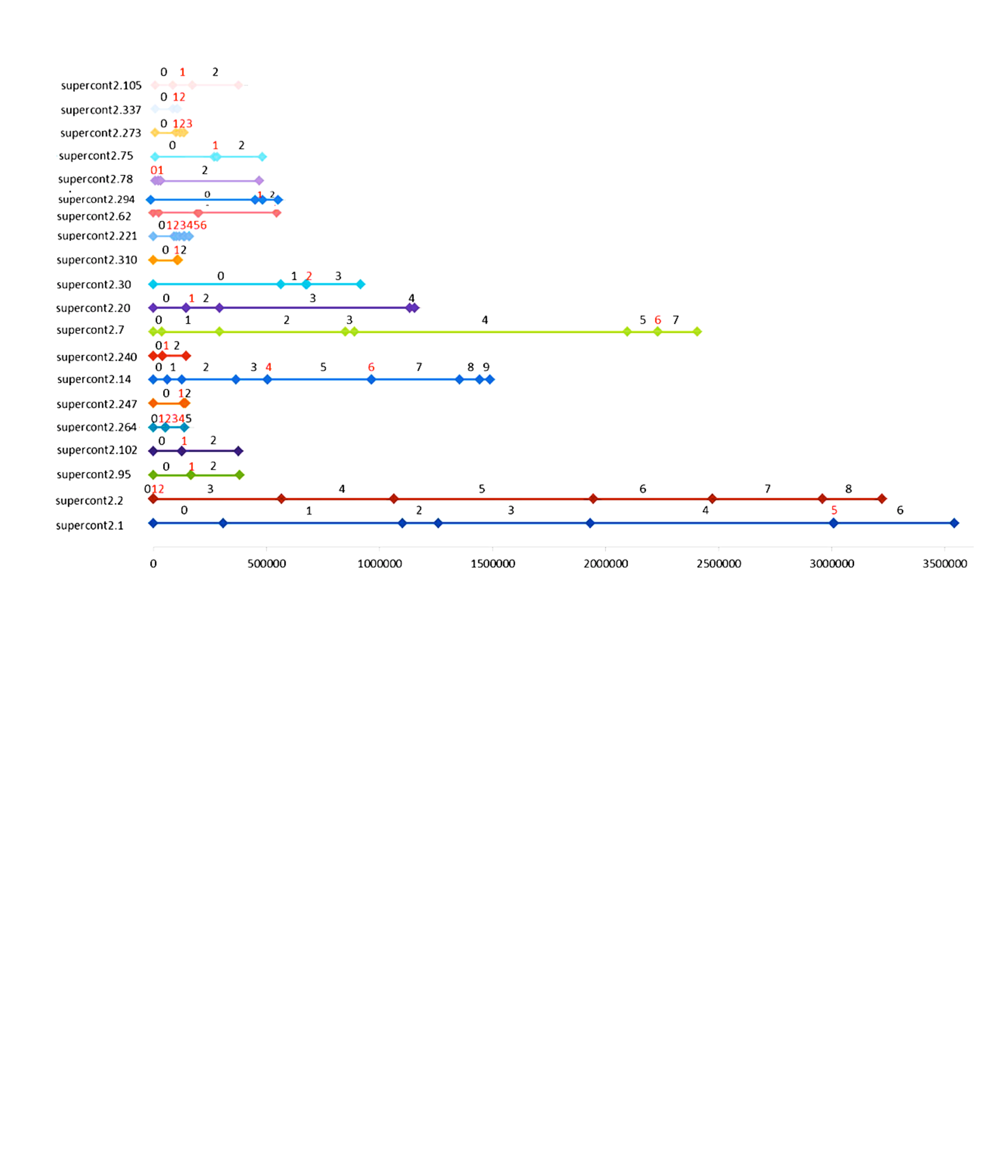

Supplement: S3 Fig — Each line indicates a different MIC supercontig, and the numbering method is identical to that in Fig 2A. The red letter indicates the Cbs-bounded section that is eliminated from the MAC genome. (TIF) [file pgen.1006403.s003.tif]

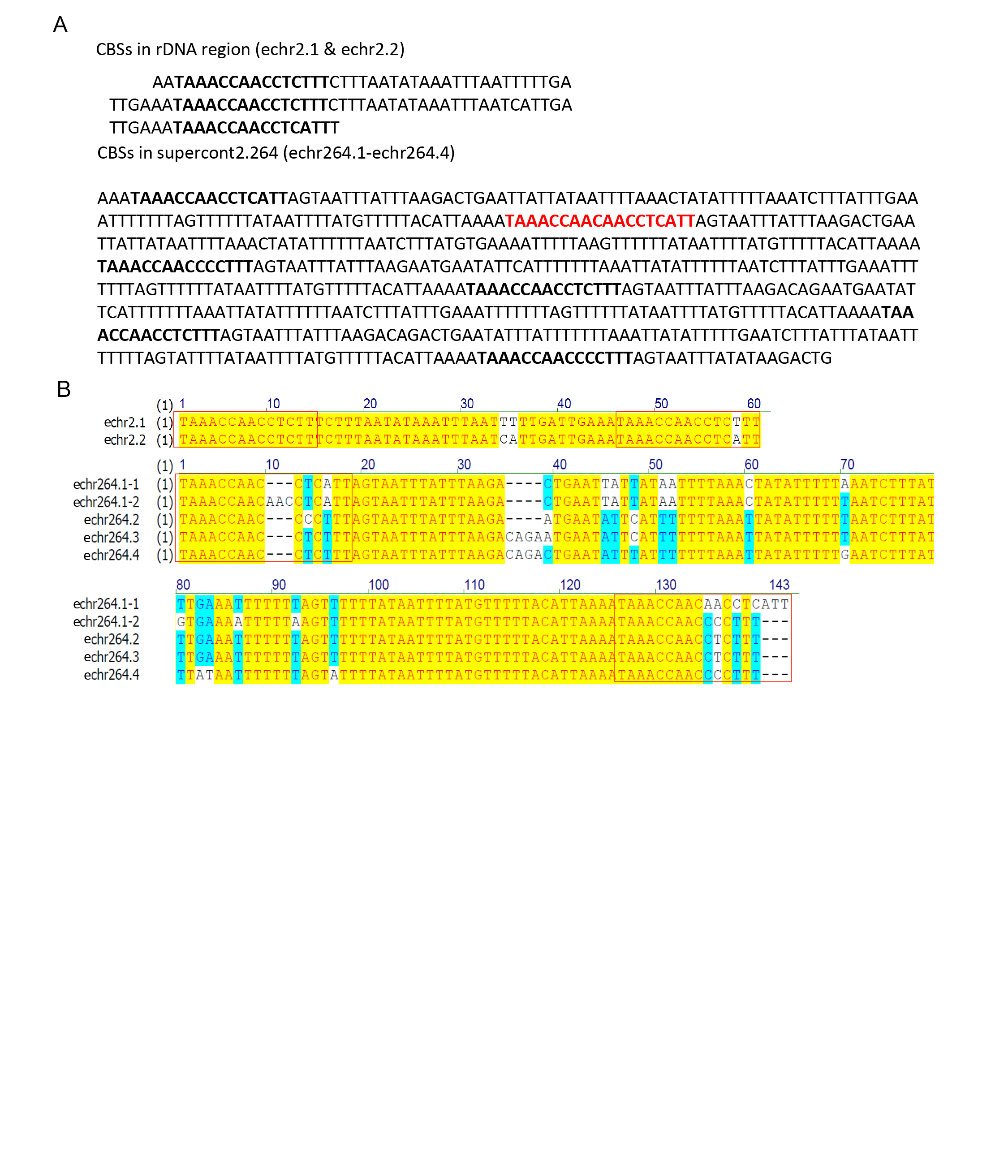

Supplement: S4 Fig — (A) Two eliminated minichromosomes show tandem repeats that are bounded by Cbs sites. Black bold letters: Cbs; Red bold letters: degenerate Cbs. (B) Sequence comparison of the repeats shown in (A). (TIF) [file pgen.1006403.s004.tif]

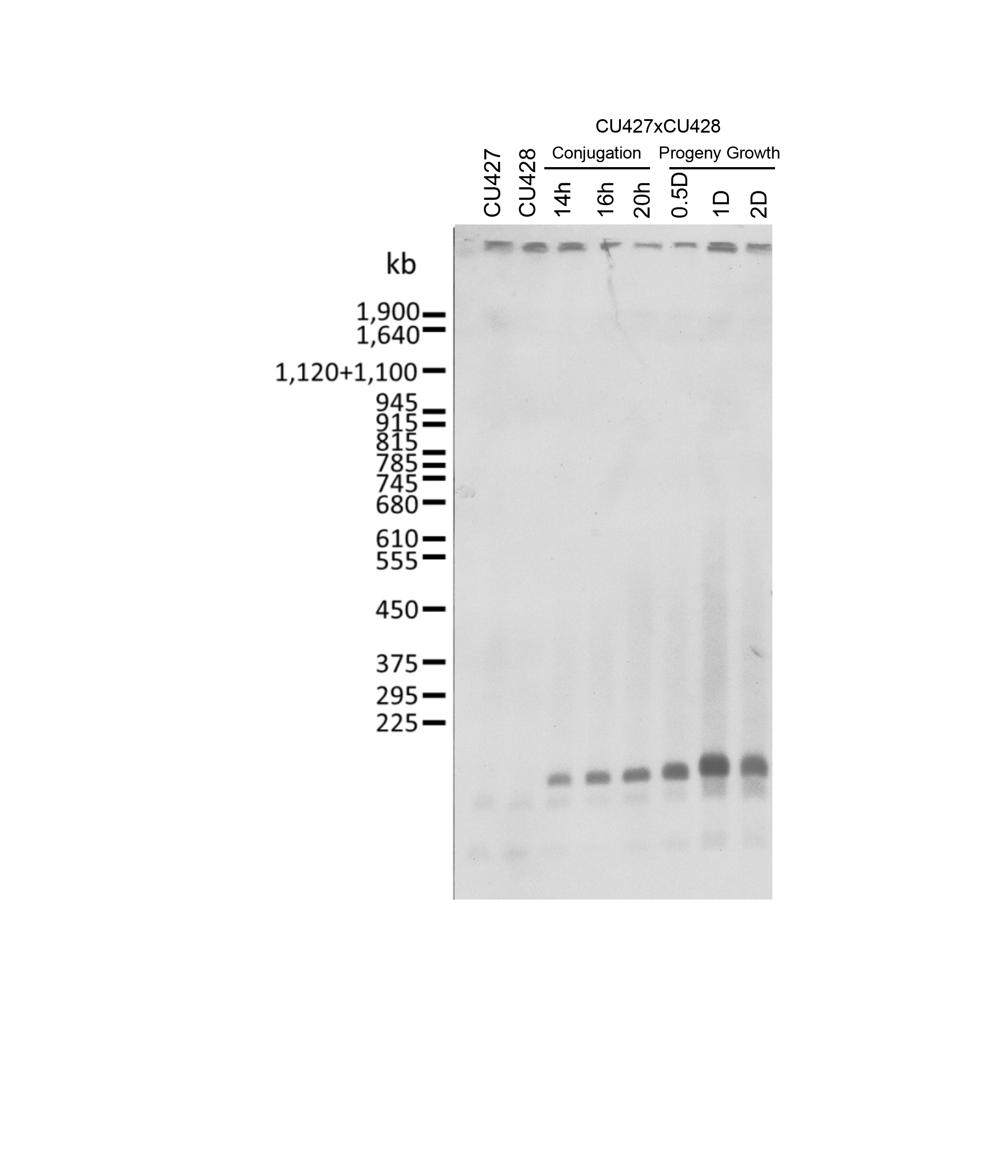

Supplement: S5 Fig — Uncut whole cell DNA samples were collected at different time points (hours after mixing cells for mating and hours or doubling after conjugation) and separated in an agarose gel by pulsed-field gel electrophoresis. The doubling time is about 3 hours in growing population. (TIF) [file pgen.1006403.s005.tif]

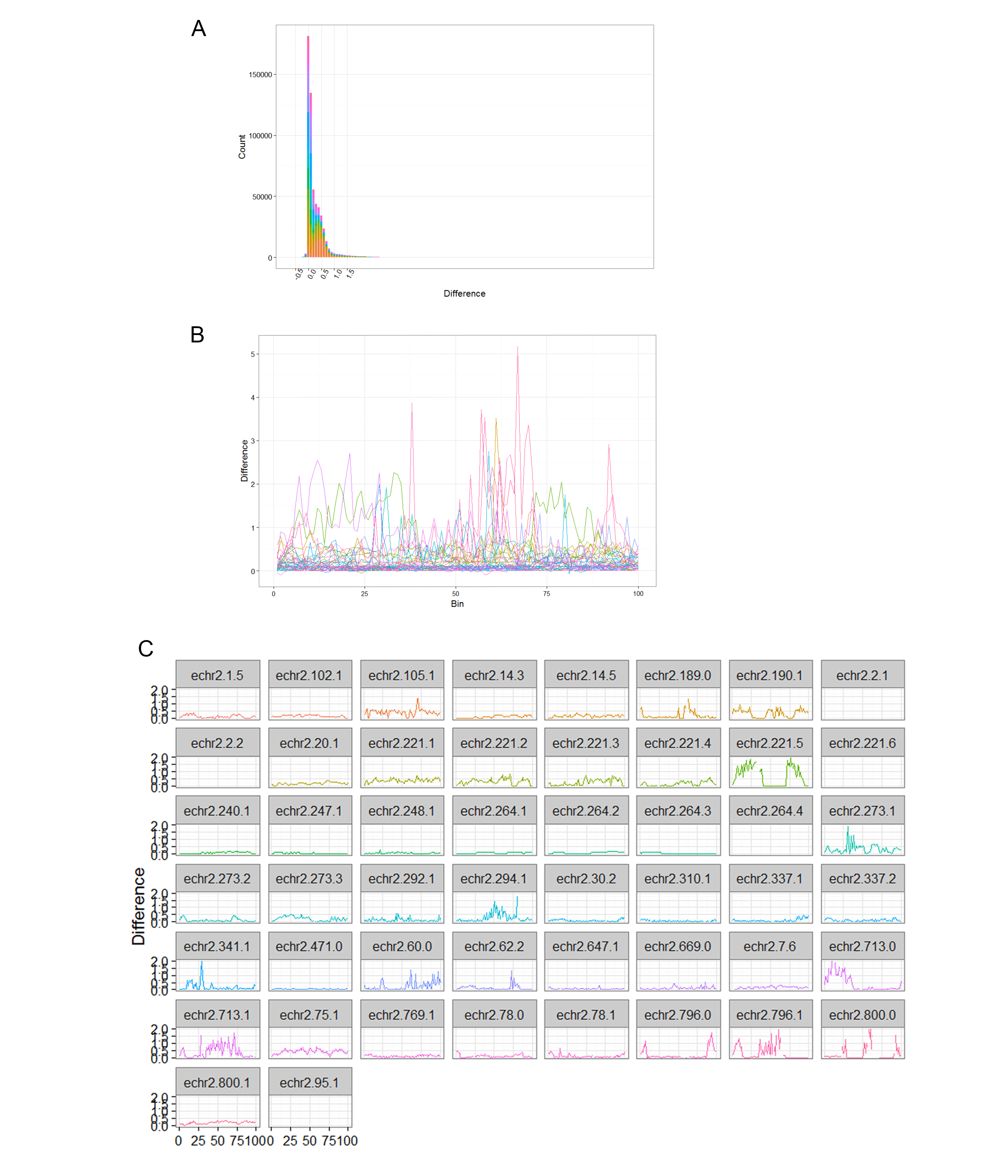

Supplement: S6 Fig — (A) Accumulated Frequency of DNA coverage differences. 56.5% of the counts show in the difference less than 0.1, indicating the region with low coverage. (B) and (C) Distribution of differences for EMCs. X-axis indicates the percentage of length. Each EMC is divided into 100 bins, and the average difference of each bin is shown. (TIF) [file pgen.1006403.s006.tif]

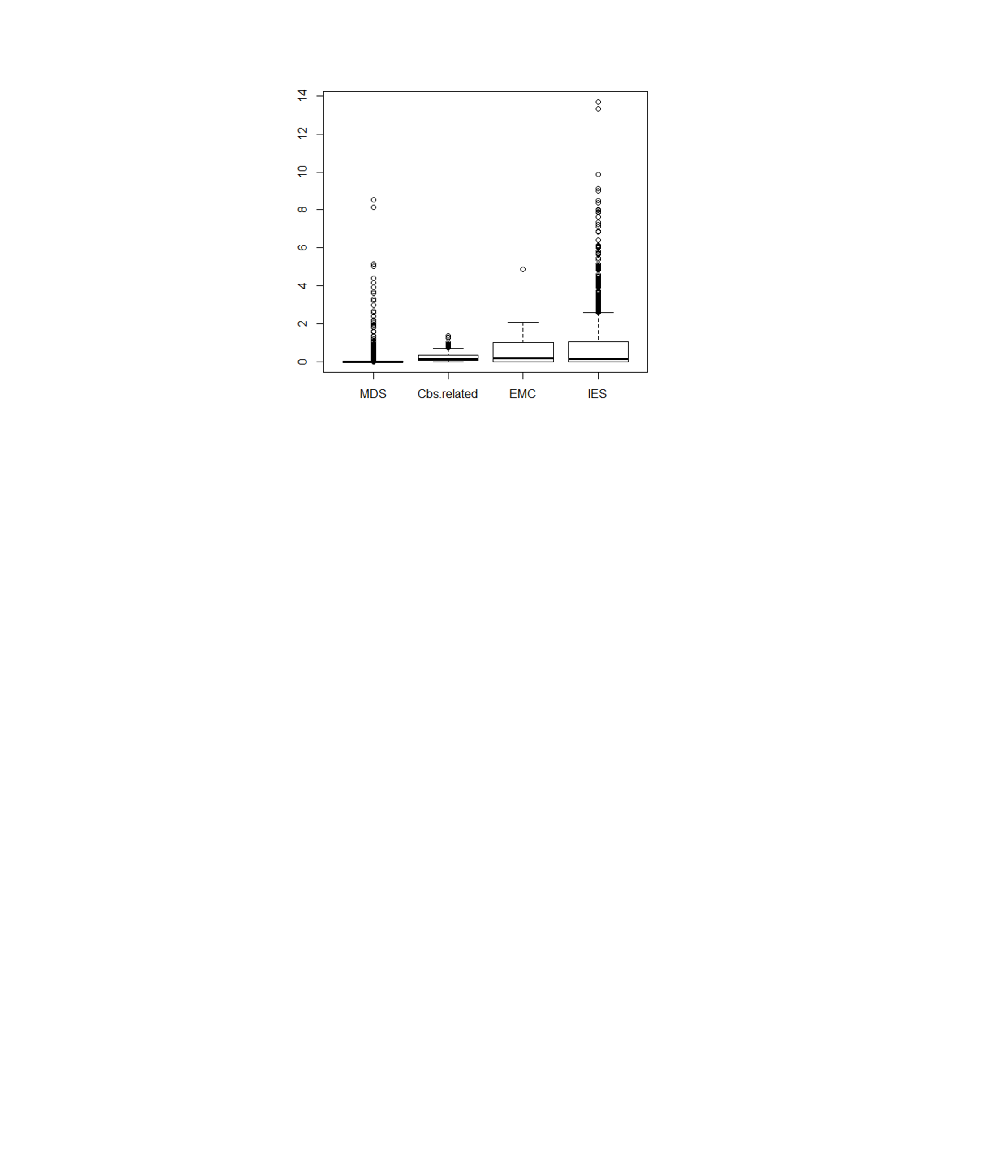

Supplement: S7 Fig — The amount of fold enrichment of MDS, Cbs-related section, EMC and IES is 0.04, 0.23, 0.60 and 0.68, respectively. MDS: MAC-destine sequence; Cbs-related: Cbs-relate section; EMC: eliminated minichromosome; IES: internal eliminated sequence. (TIF) [file pgen.1006403.s007.tif]

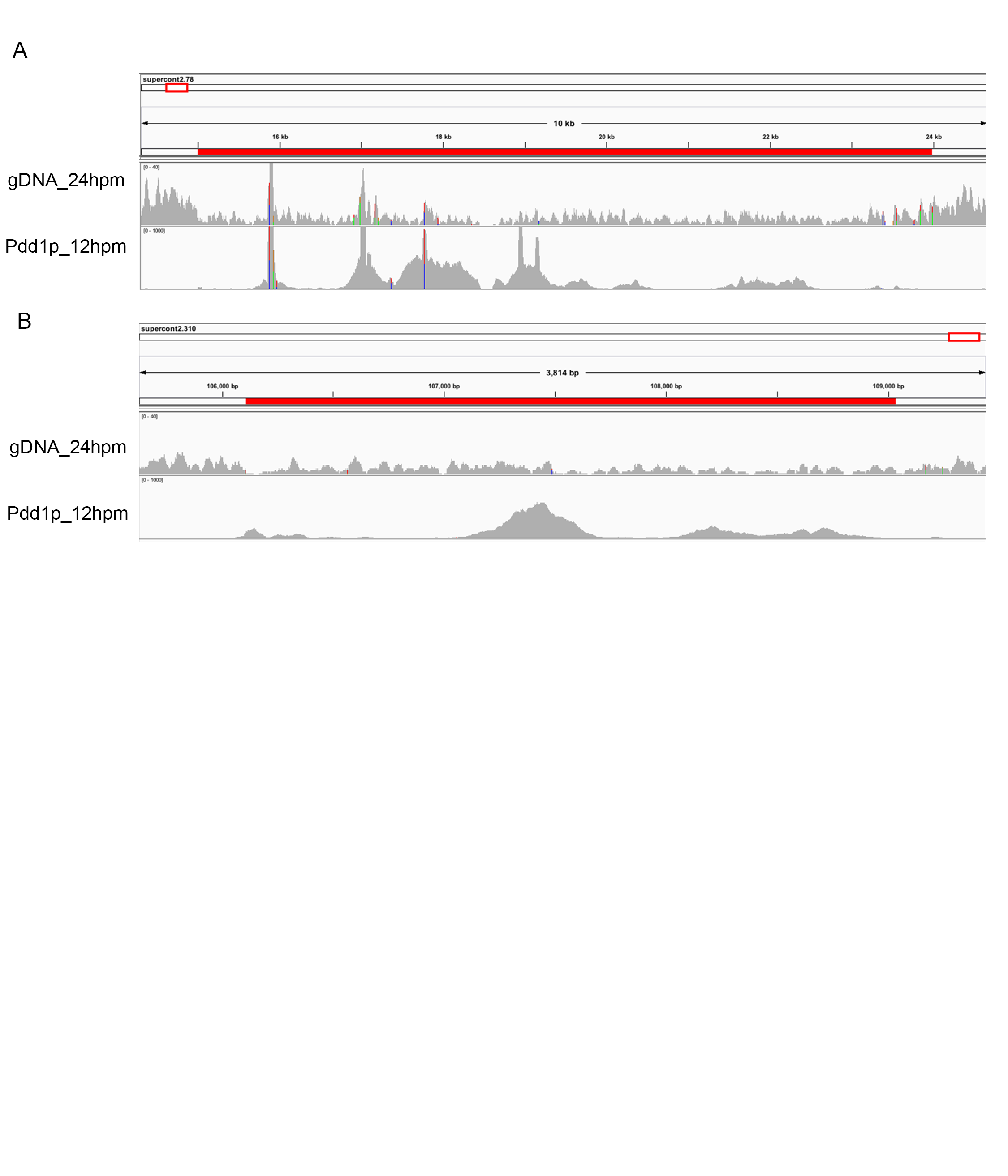

Supplement: S8 Fig — The read coverage of gDNA and the Pdd1p-bound region at echr2.78.1 (A) and echr2.310.1 (B). The red boxes indicate the region of IES. (TIF) [file pgen.1006403.s008.tif]

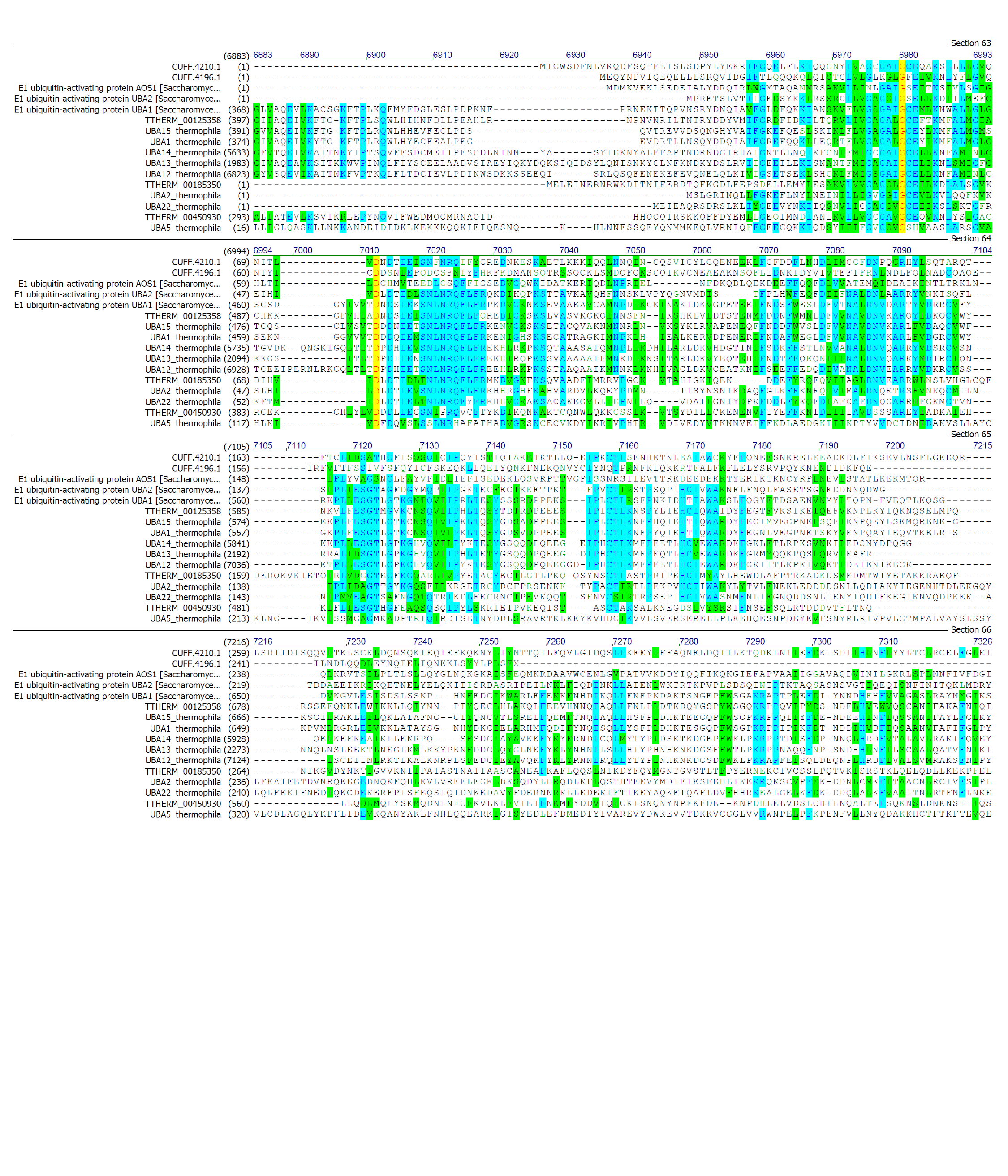

Supplement: S9 Fig — CUFF.4210.1 indicates the E1-like protein 1 and CUFF.4196.1 indicates the other E1-like gene identified in this study. (TIF) [file pgen.1006403.s009.tif]

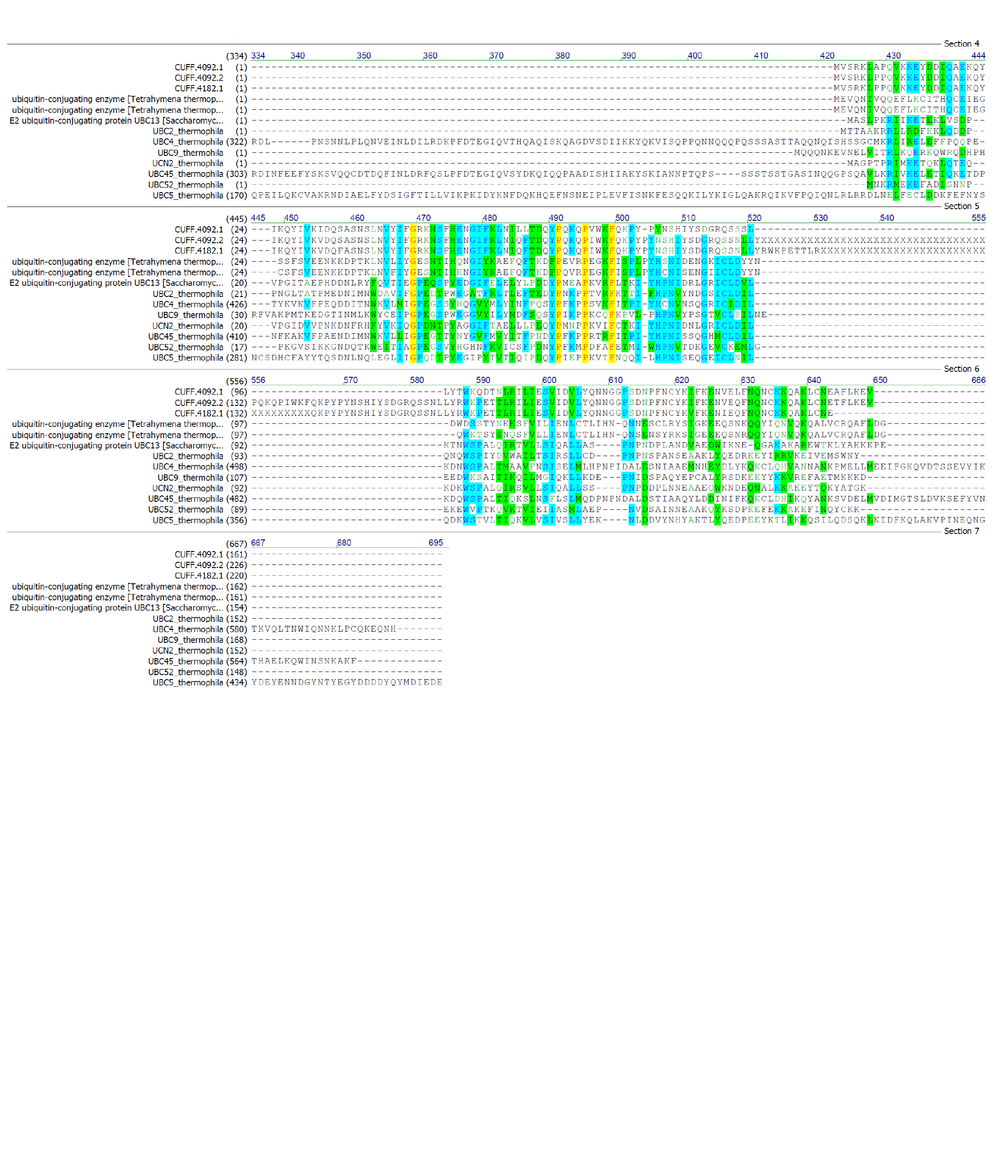

Supplement: S10 Fig — CUFF.4092.1 indicates the E2-like protein 1, CUFF.4092.2 and CUFF.4182.1 indicate other E2-like genes identified in this study. (TIF) [file pgen.1006403.s010.tif]

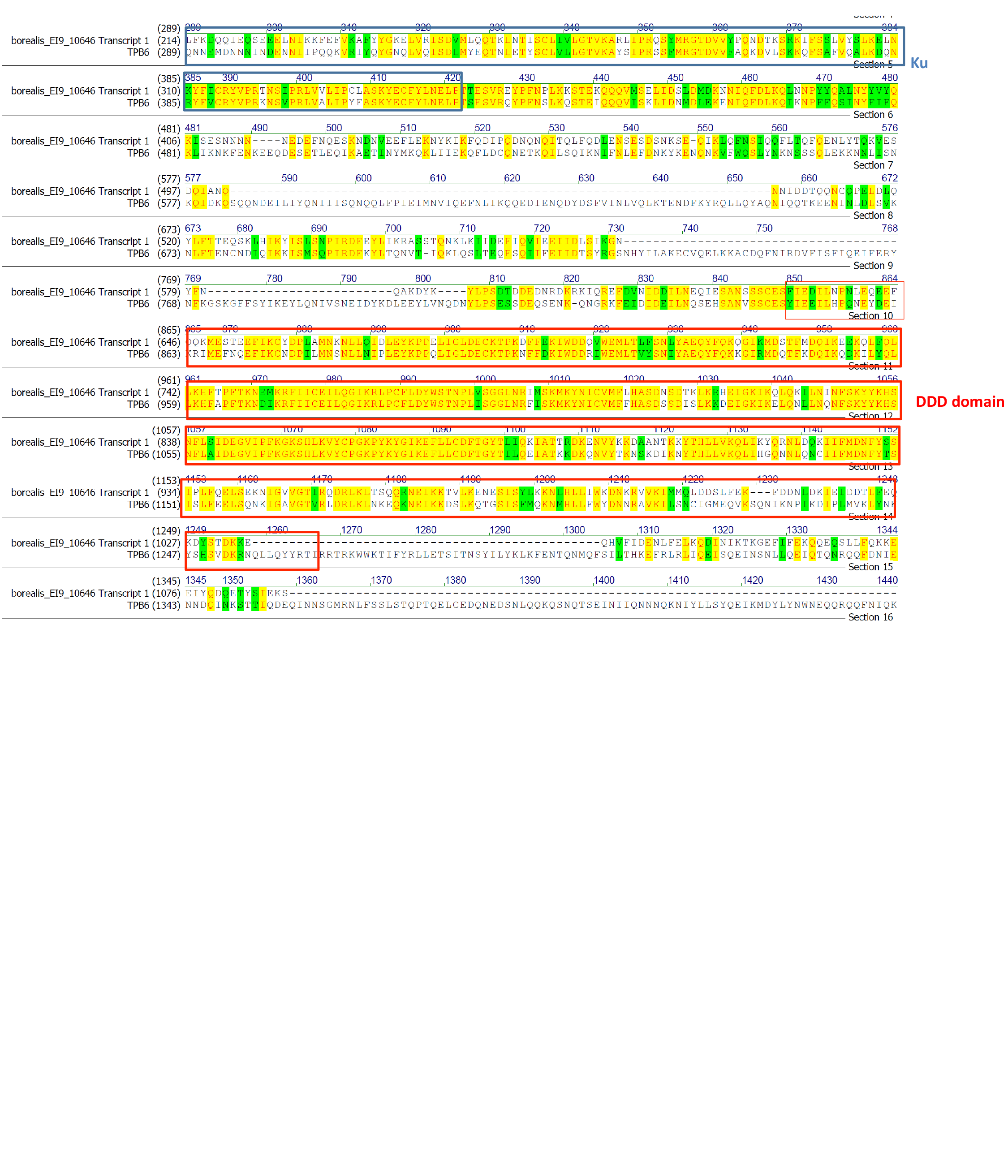

Supplement: S11 Fig — Blue box: the conserved Ku domain. Red box: the catalytic DDD domain of piggyBac transposase. (TIF) [file pgen.1006403.s011.tif]
