# Supplementary material for: Programmed Minichromosome Elimination as a Mechanism for Somatic Genome Reduction in Tetrahymena thermophila
Source: PLoS Genet. 2016 Nov 2;12(11):e1006403. doi: 10.1371/journal.pgen.1006403 (PMC5091840; doi:10.1371/journal.pgen.1006403)
Supplement: S1 Table — (DOCX) [file pgen.1006403.s012.docx]

**S1 Table. The frequency of nucleotide substitutions in each position of Cbs.**

| Mis | A | A | A | **G** | A | **G** | **G** | **T** | **T** | **G** | **G** | **T** | **T** | **T** | A | No. |
| --- | --- | --- | --- | --- | --- | --- | --- | --- | --- | --- | --- | --- | --- | --- | --- | --- |
| 0 |  |  |  |  |  |  |  |  |  |  |  |  |  |  |  | 95 |
| 1 | T | **-** | **-** | **-** | **-** | **-** | **-** | **-** | **-** | **-** | **-** | **-** | **-** | **-** | **-** | 9 |
| 1 | **-** | T | **-** | **-** | **-** | **-** | **-** | **-** | **-** | **-** | **-** | **-** | **-** | **-** | **-** | 9 |
| 1 | **-** | G | **-** | **-** | **-** | **-** | **-** | **-** | **-** | **-** | **-** | **-** | **-** | **-** | **-** | 4 |
| 1 | **-** | **-** | T | **-** | **-** | **-** | **-** | **-** | **-** | **-** | **-** | **-** | **-** | **-** | **-** | 7 |
| 1 | **-** | **-** | G | **-** | **-** | **-** | **-** | **-** | **-** | **-** | **-** | **-** | **-** | **-** | **-** | 2 |
| 1 | **-** | **-** | **-** | **-** | G | **-** | **-** | **-** | **-** | **-** | **-** | **-** | **-** | **-** | **-** | 8 |
| 1 | **-** | **-** | **-** | **-** | **-** | **-** | **-** | **-** | **-** | **-** | **-** | **-** | **-** | **-** | T | 52 |
| 2 | T | T | **-** | **-** | **-** | **-** | **-** | **-** | **-** | **-** | **-** | **-** | **-** | **-** | **-** | 1 |
| 2 | T | **-** | **-** | **-** | G | **-** | **-** | **-** | **-** | **-** | **-** | **-** | **-** | **-** | **-** | 1 |
| 2 | T | **-** | **-** | **-** | **-** | **-** | **-** | **-** | **-** | **-** | **-** | **-** | **-** | **-** | T | 8 |
| 2 | **-** | G | **-** | **-** | G | **-** | **-** | **-** | **-** | **-** | **-** | **-** | **-** | **-** | **-** | 1 |
| 2 | **-** | G | **-** | **-** | **-** | **-** | **-** | **-** | **-** | **-** | **-** | **-** | **-** | **-** | T | 2 |
| 2 | **-** | **-** | C | **-** | G | **-** | **-** | **-** | **-** | **-** | **-** | **-** | **-** | **-** | **-** | 1 |
| 2 | **-** | **-** | G | **-** | **-** | **-** | **-** | **-** | **-** | **-** | **-** | **-** | **-** | **-** | T | 1 |
| 2 | **-** | **-** | T | **-** | G | **-** | **-** | **-** | **-** | **-** | **-** | **-** | **-** | **-** | **-** | 1 |
| 2 | **-** | **-** | T | **-** | **-** | **-** | **-** | **-** | **-** | **-** | **-** | **-** | **-** | **-** | T | 2 |
| 2 | **-** | **-** | **-** | **-** | G | **-** | **-** | **-** | **-** | **-** | **-** | **-** | **-** | **-** | T | 5 |
| Non-founctional Cbs-like sequences (< 3 substitution, total 896) | | | | | | | | | | | | | | | | |
| 2 |  |  |  | T |  |  |  |  |  |  |  |  |  |  | C | 51 |
| 2 |  |  |  |  |  |  |  |  |  | T |  |  |  |  | C | 34 |
| 2 |  |  |  |  |  | T |  |  |  | T |  |  |  |  |  | 17 |
| 2 |  |  |  | T |  |  |  |  |  | T |  |  |  |  |  | 13 |
| 2 |  |  |  |  |  |  | T |  |  |  | T |  |  |  |  | 13 |
| 2 |  |  |  |  |  | A |  |  |  |  | A |  |  |  |  | 11 |
| 2 |  |  |  |  |  |  |  |  |  | T |  |  |  |  | T | 10 |
| 2 |  |  |  | T |  |  |  |  |  |  | T |  |  |  |  | 10 |
| 2 |  |  |  |  |  | A |  |  |  | A |  |  |  |  |  | 10 |
| pos | 1 | 2 | 3 | 4 | 5 | 6 | 7 | 8 | 9 | 10 | 11 | 12 | 13 | 14 | 15 |  |
