# Supplementary material for: Programmed Minichromosome Elimination as a Mechanism for Somatic Genome Reduction in Tetrahymena thermophila
Source: PLoS Genet. 2016 Nov 2;12(11):e1006403. doi: 10.1371/journal.pgen.1006403 (PMC5091840; doi:10.1371/journal.pgen.1006403)
Supplement: S2 Table — (DOCX) [file pgen.1006403.s013.docx]

**S2 Table. List of eliminated minichromosomes.**

| ID | Position 1 | Position 2 | Length |
| --- | --- | --- | --- |
| echr2.2.1 | 805 | 834 | 30 |
| echr2.2.2 | 850 | 879 | 30 |
| *echr2.221.6 | 160,011 | 160,041 | 31 |
| echr2.95.1 | 166,219 | 166,287 | 69 |
| echr2.264.4 | 54,882 | 54,961 | 80 |
| echr2.264.2 | 54,642 | 54,744 | 103 |
| echr2.264.3 | 54,760 | 54,866 | 107 |
| echr2.264.1 | 54,403 | 54,626 | 224 |
| echr2.247.1 | 135,661 | 135,974 | 314 |
| *echr2.800.1 | 12,085 | 12,525 | 441 |
| echr2.14.3 | 506,354 | 506,807 | 454 |
| echr2.102.1 | 127,960 | 128,474 | 515 |
| echr2.240.1 | 40,794 | 41,383 | 590 |
| echr2.7.6 | 2,230,722 | 2,231,362 | 641 |
| echr2.20.1 | 146,067 | 146,847 | 781 |
| *echr2.471.0 | 1 | 878 | 878 |
| echr2.14.5 | 965,452 | 966,559 | 1,108 |
| *echr2.273.3 | 125,947 | 128,200 | 2,254 |
| echr2.1.5 | 3,008,999 | 3,011,420 | 2,422 |
| *echr2.796.0 | 1 | 2,988 | 2,988 |
| *echr2.713.0 | 1 | 3,218 | 3,218 |
| echr2.30.2 | 676,495 | 679,861 | 3,367 |
| echr2.310.1 | 105,639 | 109,443 | 3,805 |
| *echr2.337.2 | 96,328 | 101,506 | 5,179 |
| *echr2.769.1 | 8,864 | 14,423 | 5,560 |
| echr2.221.4 | 135,426 | 141,051 | 5,626 |
| echr2.62.2 | 196,702 | 203,450 | 6,769 |
| *echr2.292.1 | 110,878 | 117,701 | 6,824 |
| echr2.294.1 | 96,065 | 103,014 | 6,950 |
| *echr2.796.1 | 3,004 | 12,625 | 9,622 |
| echr2.78.1 | 14,315 | 24,674 | 10,360 |
| echr2.221.1 | 91,883 | 103,321 | 11,439 |
| echr2.75.1 | 261,980 | 273,701 | 11,722 |
| *echr2.800.0 | 1 | 12,068 | 12,068 |
| echr2.221.2 | 103,337 | 115,991 | 12,655 |
| *echr2.78.0 | 1 | 14,299 | 14,299 |
| echr2.273.2 | 110,111 | 125,931 | 15,821 |
| echr2.337.1 | 80,058 | 96,312 | 16,255 |
| *echr2.713.1 | 3,234 | 19,545 | 16,312 |
| echr2.669.0 | 1 | 18,032 | 18,032 |
| echr2.221.5 | 141,067 | 159,995 | 18,929 |
| echr2.273.1 | 90,996 | 110,095 | 19,100 |
| echr2.221.3 | 116,007 | 135,410 | 19,404 |
| *echr2.189.0 | 1 | 20,007 | 20,007 |
| *echr2.647.1 | 4,295 | 26,621 | 22,327 |
| *echr2.248.1 | 110,980 | 142,508 | 31,529 |
| *echr2.60.0 | 1 | 33,825 | 33,825 |
| *echr2.190.1 | 131,254 | 183,427 | 52,174 |
| *echr2.341.1 | 49,478 | 103,290 | 53,813 |
| echr2.105.1 | 79,831 | 163,617 | 83,787 |

*Minichromosome that was bounded by Cbs and an end of a supercontig.
