# Supplementary material for: Programmed Minichromosome Elimination as a Mechanism for Somatic Genome Reduction in Tetrahymena thermophila
Source: PLoS Genet. 2016 Nov 2;12(11):e1006403. doi: 10.1371/journal.pgen.1006403 (PMC5091840; doi:10.1371/journal.pgen.1006403)
Supplement: S3 Table — (DOCX) [file pgen.1006403.s014.docx]

**S3 Table. The fold enrichment of Pdd1p in EMCs.**

| ID | Pdd1p fold |
| --- | --- |
| supercont2.221.6 | 0 |
| supercont2.2.1 | 0 |
| supercont2.2.2 | 0 |
| supercont2.95.1 | 0 |
| supercont2.264.4 | 0 |
| supercont2.264.2 | 0 |
| supercont2.264.3 | 0 |
| supercont2.264.1 | 0 |
| supercont2.247.1 | 0 |
| supercont2.471.0 | 0 |
| supercont2.800.1 | 0 |
| supercont2.14.3 | 0 |
| supercont2.102.1 | 0 |
| supercont2.7.6 | 0 |
| supercont2.20.1 | 0 |
| supercont2.14.5 | 0 |
| supercont2.796.0 | 0 |
| supercont2.30.2 | 0 |
| supercont2.221.1 | 0 |
| supercont2.75.1 | 0 |
| supercont2.221.2 | 0 |
| supercont2.221.4 | 0.08 |
| supercont2.190.1 | 0.12 |
| supercont2.240.1 | 0.14 |
| supercont2.273.3 | 0.17 |
| supercont2.221.5 | 0.18 |
| supercont2.105.1 | 0.18 |
| supercont2.221.3 | 0.27 |
| supercont2.713.0 | 0.3 |
| supercont2.713.1 | 0.37 |
| supercont2.1.5 | 0.54 |
| supercont2.273.1 | 0.54 |
| supercont2.62.2 | 0.62 |
| supercont2.337.1 | 0.71 |
| supercont2.796.1 | 0.77 |
| supercont2.647.1 | 0.9 |
| supercont2.78.0 | 1 |
| supercont2.310.1 | 1.01 |
| supercont2.78.1 | 1.01 |
| supercont2.294.1 | 1.08 |
| supercont2.341.1 | 1.08 |
| supercont2.800.0 | 1.1 |
| supercont2.273.2 | 1.1 |
| supercont2.189.0 | 1.3 |
| supercont2.248.1 | 1.71 |
| supercont2.60.0 | 1.78 |
| supercont2.669.0 | 1.79 |
| supercont2.292.1 | 1.94 |
| supercont2.337.2 | 2.09 |
| supercont2.769.1 | 4.87 |
