# Supplementary material for: Programmed Minichromosome Elimination as a Mechanism for Somatic Genome Reduction in Tetrahymena thermophila
Source: PLoS Genet. 2016 Nov 2;12(11):e1006403. doi: 10.1371/journal.pgen.1006403 (PMC5091840; doi:10.1371/journal.pgen.1006403)
Supplement: S4 Table — (DOCX) [file pgen.1006403.s015.docx]

**S4 Table. The conserved genes in other *Tetrahymena* species.**

| Species | Contig ID of match species | echr ID | transcript ID in *T. thermophila* | Identity (%) | Matched length (a.a) |
| --- | --- | --- | --- | --- | --- |
| *borealis* | Supercontig_1.55 | echr2.105.1 | *CUFF.4075.1* | 47.93 | 507 |
| *borealis* | Supercontig_1.55 | echr2.105.1 | *CUFF.4076.1* | 47.23 | 506 |
| *elliotti* | Supercontig_2.83 | echr2.105.1 | *CUFF.4075.1* | 65.21 | 503 |
| *elliotti* | Supercontig_2.83 | echr2.105.1 | *CUFF.4076.1* | 66.2 | 503 |
| *elliotti* | Supercontig_2.83 | echr2.105.1 | *CUFF.4080.1* | 56.07 | 2299 |
| *elliotti* | Supercontig_2.83 | echr2.105.1 | *CUFF.4102.1* | 56.82 | 2323 |
| *elliotti* | Supercontig_2.6 | echr2.105.1 | *CUFF.4092.1* | 52.34 | 107 |
| *borealis* | Supercontig_1.78 | echr2.105.1 | *CUFF.4095.1* | 49.03 | 155 |
| *borealis* | Supercontig_1.78 | echr2.105.1 | *CUFF.4108.1* | 49.4 | 166 |
| *borealis* | Supercontig_1.78 | echr2.105.1 | *CUFF.4111.1* | 41.58 | 190 |
| *borealis* | Supercontig_1.78 | echr2.105.1 | *CUFF.4125.1* | 44.52 | 155 |
| *borealis* | Supercontig_1.78 | echr2.105.1 | *CUFF.4137.1* | 48.65 | 148 |
| *borealis* | Supercontig_1.78 | echr2.105.1 | *CUFF.4196.1* | 51.11 | 270 |
| *borealis* | Supercontig_1.78 | echr2.105.1 | *CUFF.4210.1* | 44.14 | 367 |
| *borealis* | Supercontig_1.46 | echr2.75.1 | *CUFF.25882.1* | 52.07 | 386 |
