# Supplementary material for: Programmed Minichromosome Elimination as a Mechanism for Somatic Genome Reduction in Tetrahymena thermophila
Source: PLoS Genet. 2016 Nov 2;12(11):e1006403. doi: 10.1371/journal.pgen.1006403 (PMC5091840; doi:10.1371/journal.pgen.1006403)
Supplement: S5 Table — (DOCX) [file pgen.1006403.s016.docx]

| **Southern probe** |  |
| --- | --- |
| 2.105.1-F | ATGAGTTATAGATATAGAGTAG |
| 2.105.1-R | AAGCTAAATAATATTAATCTTGA |
| 2.75.1-F | AACTTTCGTCTTCTATCAAG |
| 2.75.1-R | ACAATTCTGGTATGCGTAAT |
| DRB3-F | GGTACC GACATTTAGTACACTTTGCGTTTG |
| DRB3-R | GGATCC CTTATTTTAATTGTTTTATAGGATCGC |
| **IES junction** |  |
| 2.310.1-ies-F | GTCAAGCTACCTAGTAAAATTG |
| 2.310.1-ies-R1 | AAGTGCCTAATGAAATATAGCC |
| 2.78.1-ies-F | AGAATTACAACCACTTTAATCATACAT |
| 2.78.1-ies-R | GTGAGGAAGGATGATATTGATTG |
| **quantitative-PCR** |  |
| M element-F | AGCTTAAACAAATGCATATTGAG |
| M-element-R | GTGGGGAGGGAGAAGGATTCAAC |
| ATU1-F | CACTGGTTTCAAGGTCGGTAT |
| ATU1-R | TCAGTAACCTTCTTCTTCTTCACC |
| TPB6-qpcr-F | GTAATTGAAGTTTCGAGAAG |
| TPB6-qpcr-R | GGTATGATAATAACAGAGCTGT |
| E2-like1-F | CTGCCTCAAATTCTTTAAAT |
| E2-like1-R | GCGAGTTATATGGATAAGGT |
| E1-like1-F | AGATAGCGCTACTCATGGAT |
| E1-like1-R | GCCTCTAAGTTTGTCTTATG |
| **Telomere-anchored PCR** | |
| Telomere | CCCCAACCCCAACCCCAA |
| 2.240.1-3'telo | GCATCTTTAATATATCTACATAC |
| 2.310.1-3'telo | CGGCCATTTGGCTATATTTC |
| 2.247.1-5'telo | GATACTTTAGCTCTAGCTCTATTTC |
| 2.75.1-5'telo | CAAAGCGAAAATAATATCAG |
| 2.78.1-3'telo | CAATCAATATCATCCTTCCTCAC |
| 2.1.5-L-5’teloR | CTTACCCAACATACACATTTCTTACT |
| 2.105.1-83803-telo-F | GCGAAAGTTTAAGTGTTCCCTTAAG |
| 2.190.1-L-5’teloR | TATTTATGATGTAACGCAGGCA |
| 2.294.1-L-3’teloF | GGCGTTGCTTAGAAGTAAGTG |
| 2.102.1-L-5’teloR | TAGAACAAGACCACCTTTATGG |
| Cbs819-503 | GATCAAACTGAGTACTCACTATC |
| ATU1-F | CACTGGTTTCAAGGTCGGTAT |
| ATU1-R | TCAGTAACCTTCTTCTTCACC |
