# Supplementary material for: Programmed Minichromosome Elimination as a Mechanism for Somatic Genome Reduction in Tetrahymena thermophila
Source: PLoS Genet. 2016 Nov 2;12(11):e1006403. doi: 10.1371/journal.pgen.1006403 (PMC5091840; doi:10.1371/journal.pgen.1006403)
Supplement: S1 Text — (DOCX) [file pgen.1006403.s017.docx]

**Material and methods**

**Chromatin immunoprecipitation sequencing analysis**

The published dataset was sourced from the gene expression omnibus (GEO). Raw sequencing reads that were download from GSE70083 are ChIP-seq analysis using the anti-Pdd1p antibody from wild-type new MACs (GSM1716851) and input for ChIP from wild-type new MACs (the first sample, GSM1716850). The MIC genome assembly data serves as the reference for sequence alignment [56] using bowtie2 [59] and SAM/BAM file handling was done by SAMtools [60]. The mapped reads were used to detect peaks using MACS2 (p-value > 0.001 and FDR < 0.001) and generate the fold enrichment file by comparing the sample to the input [68].

**Pulsed-field gel electrophoresis**

Uncut total DNAs were prepared as previously described [69, 70] with several modifications. In brief, samples (5$\times$10^5^ cells/plug) from starved and different conjugation time points were washed in 10 mM Tris buffer (pH 8.0) and mixed with 1.6% low melting agarose (Sigma-Aldrich Corp.). The samples were poured into a casting mould (Biometra GmbH, Göttingen Germany) and kept for 10 min at 4°C to solidify. The agarose plugs were incubated in the lysis solution (10 mM Tris buffer [pH 8.0], 0.5M EDTA, 1% SDS, and 1 mg/ml proteinase K) overnight at 42°C. The agarose plugs were washed 3$\times$1 h in TE buffer at room temperature and maintained in TE buffer (pH 8.0) at 4°C. Subsequently, the agarose plugs were inserted into the well of a 0.8% gel to perform pulsed-field gel electrophoresis (0.5X TBE, interval time of 50 sec, angle 110°, and 100V for 78 h) in Rotaphor® System 6.0, Biometra.

**Reference**

68. Zhang Y, Liu T, Meyer CA, Eeckhoute J, Johnson DS, Bernstein BE, et al. Model-based analysis of ChIP-Seq (MACS). Genome biology. 2008;9(9):R137. DOI: 10.1186/gb-2008-9-9-r137.

69. Schwartz DC, Cantor CR. Separation of yeast chromosome-sized DNAs by pulsed field gradient gel electrophoresis. Cell. 1984;37(1):67-75. <http://www.ncbi.nlm.nih.gov/pubmed/6373014>

70. Lukaszewicz A, Howard-Till RA, Novatchkova M, Mochizuki K, Loidl J. MRE11 and COM1/SAE2 are required for double-strand break repair and efficient chromosome pairing during meiosis of the protist *Tetrahymena*. Chromosoma. 2010;119(5):505-18. DOI: 10.1007/s00412-010-0274-9.
